# An application of *hp*-version finite element methods to quench simulation in axisymmetric MRI magnets

**DOI:** 10.1007/s00366-025-02126-y

**Published:** 2025-04-05

**Authors:** M. S. Miah, P. D. Ledger, A. J. Gil, M. Mallett, T.-Q. Ye

**Affiliations:** 1 School of Computer Science & Mathematics, Keele University, Keele, ST5 5BG UK; 2 School of Computing & Mathematical Sciences, University of Leicester, Leicester, LE1 7RH UK; 3 Faculty of Science and Engineering, Zienkiewicz Institute for Modelling, Data and AI, Swansea University, Swansea, SA1 8EN UK; 4 MR Magnet Technology, Siemens Healthineers, Eynsham, OX29 4BP UK

**Keywords:** Magnet quench, Superconductivity, Coupled physics problem, Magnetic Resonance Imaging, *hp*-Version finite element method

## Abstract

Magnetic Resonance Imaging (MRI) scanners employ superconducting magnets to produce a strong uniform magnetic field over the bore of the scanner as part of the imaging process. Superconductors are preferred, as they can generate the required field strengths without electrical resistance, but, to do this, the materials need to be cooled to very low temperatures, typically around 4.2 K. However, due to imperfections in the windings, cracks and small air gaps in the epoxy resin between the wires, heating can occur leading to a process known as magnet quench. During magnet quench, the magnet temperature rises quickly, and the magnet loses its superconductivity. This work presents an accurate numerical model for predicting magnet quench for axisymmetric MRI scanners by solving the coupled system of thermal, electromagnetic and circuit equations by means of a high order/*hp*-version finite element method where regions of high gradients are resolved with boundary layer elements. A series of numerical results are included to demonstrate the effectiveness of the approach.

## Introduction

The use of Magnetic Resonance Imaging (MRI) has been an essential tool in modern medical diagnosis with use cases ranging from the examination of the brain and spinal cord for tumours to internal organs such as the liver or the prostate gland. Our group’s previous work involved the simulation of a coupled 3D magneto mechanical problem to predict resonance effects associated with vibrations induced by eddy currents in conducting components (see [18] and references therein). The present work focuses on the modelling of superconducting materials used to produce the strong static magnetic field and how they transition to become normal conducting. During normal operation of an MRI scanner, the coils of superconducting material are kept cool in a bath of liquid helium so that they conduct electricity without resistance. However, due to imperfections in the windings, cracks and small air gaps in the epoxy resin between the wires, heating of the coil can occur so that the material becomes normal conducting and resistive. This process is called magnet quench. The heating of the coil boils-off the liquid helium (He), which is costly to replace. To better understand magnet quench, and predict when it is likely to occur, the solution of a highly non-linear coupled multi-physics problem involving electromagnetic, thermal and mechanical effects is required. The purpose of this paper is to present an accurate numerical scheme for resolving transient electro-thermal coupling within rotationally symmetric scanners, in addition to coupling with a circuit model to describe other components.

The textbook by Wilson [30] describes a mathematical model of quench and presents a semi-analytical approach to predicting quench. More recently, approaches have focused on solving coupled sets of thermal-electromagnetic models combined with circuit models for describing quench behaviour [21]. One of the key challenges is the modelling of the wires and strands that make up the superconducting magnets. Schöps, De Gersem and Weiland [25] consider the coupling between circuit and electromagnetic equations through winding functions that can be used for modelling such stranded conductors.

In the papers by Cortes Garcia, Schöps, Maciejewski, Bertot, Prioli, Auchmann, and Verweij [5, 11] and in the thesis by Cortes Garcia [7] an approach using the stranded conductor model and coupling electromagnetic and thermal equations is undertaken. This is simulated by low-order finite elements coupled with a circuit model and is applied to quench problems concerning superconducting magnets within the CERN particle accelerator. Furthermore, Paudel [19], similar to the approach described by Russenchuck [21], models temperature regimes where part of the current is shared by the superconducting niobium-titanium (NbTi) and the conducting copper in the cable. In addition, Aird et al. [3], considers the thermal isolation of the coils due to the existence of liquid Helium at 4.2 K in the surroundings with thermal conductivities much higher than that of the superconducting coil. Our work builds on these important developments, but considers an alternative finite element discretisation allowing for increased accuracy of quench simulations and applies it to applications relevant for quench simulation in MRI magnets. We choose to directly model the current, rather than use a current sharing approach, and consider only the He-I regime of helium due to the normal operating temperature of the magnets we will consider.

In particular, we consider higher order/*hp*-version finite element approaches applied to axisymmetric quench problems. This particular dicscretisation offers the possibility for refinement of both the mesh as well as the polynomial order of the elements in order to establish accurate solutions [9, 29] for both smooth solutions and solutions with high field gradients. Adaptive versions of *hp*-version finite elements (e.g. [8, 9]) use a-posteriori error estimators, (see for example [1] for a review of approaches) (or error indicators) to identify regions of highest error and determine whether to refine to perform a mesh (*h*-) refinement or a polynomial (*p*-) enrichment, an example of which is provided in [2]. Our algorithmic developments are built on the NGSolve finite element library [22, 23], which uses the particular set of basis functions proposed by Zaglmayr and Schöberl [24]. In addition, we make use of quadrilateral layers for resolving the steep solution gradients associated with the thermal problem and the heating of the coils as well as resolving the thin skin effects associated with the conducting bodies.

The novelties of the work are as follows:

- A review of the key governing equations and presentation of a mathematical model for modelling quench effects using the stranded conductor model.
- A new application of *hp*-version finite elements and thin quadrilateral elements for resolving boundary layer effects to the simulation of magnet quench.
- The presentation of a series of numerical results, including experimental validation, that illustrate the effectiveness of the scheme.

The material is organised as follows: Sect. 2 is devoted to the mathematical modelling and introduces the key fields and circuit variables and the governing equations. This section goes on to discuss superconducting materials as well as the coils, cables and wires used in superconducting magnets in addition to the different relevant loss terms. Sect.   3 presents the transmission problems obtained from the model presented in the previous section. Sect.  4 presents the weak variational statements at a continuous level that form the basis for finite element discretisation in Sect. 5, which also presents the chosen temporal discretisation and fixed point algorithm for treatment of the non-linearity in the solution. Section 5 also discusses the computational approach using the NGSolve finite element library. Section 6 presents a series of results to demonstrate the effectiveness of the proposed approach, firstly, for single physics problems and then, secondly, examples of the fully coupled quench problem. The paper closes with some concluding remarks.

## Mathematical modelling

### List of key field and circuit variables

We denote ${\varvec{x}} =x{\varvec{e}}_x +y{\varvec{e}}_y +z{\varvec{e}}_z$ as the position vector in terms of Cartesian coordinates and ${\varvec{x}} =r{\varvec{e}}_r +\phi {\varvec{e}}_\phi +z{\varvec{e}}_z$ as the position vector in terms of Cylindrical coordinates, where ${\varvec{e}}_x, {\varvec{e}}_y, {\varvec{e}}_z, {\varvec{e}}_r, {\varvec{e}}_\phi$ are the appropriate unit basis vectors and *t* as the time in seconds. From the electromagnetic perspective, ${\varvec{B}}({\varvec{x}},t)$ is the magnetic flux density, ${\varvec{M}}({\varvec{x}},t)$ is the magnetisation, ${\varvec{H}}({\varvec{x}},t)$ the magnetic field intensity vector, ${\varvec{E}}({\varvec{x}},t)$ the electric field intensity vector, ${\varvec{J}}({\varvec{x}},t)$ volume current density, which is split as ${\varvec{J}}({\varvec{x}},t)= {\varvec{J}}^{ext} ({\varvec{x}},t) + {\varvec{J}}^o({\varvec{x}},t)$, where ${\varvec{J}}^{ext} ({\varvec{x}},t)$ is the external current source, ${\varvec{J}}^o({\varvec{x}},t)$ is the Ohmic (or eddy) current and ${\varvec{A}}({\varvec{x}},t)$ is the magnetic vector potential. From the thermal perspective, $T({\varvec{x}},t)$ is the temperature and *P* denotes the sum of thermal sources. From the circuit perspective, *I* is the electric current, *V* is the voltage, *L* is the inductance and *R* is the resistance.

The eddy current model, which is applicable since conductivities of the shields, formers and the coils (when normal conducting) are high and the frequencies of excitation are low, is described by

1a $$\begin{aligned} {{\,\textrm{curl}\,}}{\varvec{E}} =&- \frac{\partial {\varvec{B}}}{\partial t}, \end{aligned}$$

1b $$\begin{aligned} {{\,\textrm{curl}\,}}{\varvec{H}} =&{\varvec{J}}^{ext} + {\varvec{J}}^o, \end{aligned}$$

1c $$\begin{aligned} {{\,\textrm{div}\,}}{\varvec{B}} =&0, \end{aligned}$$

1d $$\begin{aligned} {{\,\textrm{div}\,}}{\varvec{E}} =&0, \end{aligned}$$

where the constitutive laws

2a $$\begin{aligned} {\varvec{J}}^o&= \gamma {\varvec{E}} = 1/\rho ^e {\varvec{E}}, \end{aligned}$$

2b $$\begin{aligned} {\varvec{B}}&= \mu _0 ({\varvec{H}} + {\varvec{M}}), \end{aligned}$$

apply with $\gamma$ being the (non-linear) electrical conductivity, $\rho ^e$ the (non-linear) electrical resistivity and $\mu _0=4 \pi \times 10^{-7} \text {Hm}^{-1}$ is the magnetic permeability of free space. At interfaces between different materials, the interface conditions $[{\varvec{n}} \times {\varvec{E}}] = {\varvec{0}}$ and $[{\varvec{n}} \times {\varvec{H}}] = {\varvec{0}}$ apply where [] denotes the jump and ${\varvec{n}}$ denotes the unit outward normal. Initial conditions for the fields ${\varvec{E}}(t=0)={\varvec{E}}_0$ and ${\varvec{H}}(t=0)={\varvec{H}}_0$ are assumed to be known. This system is reduced by introducing the vector potential ${\varvec{A}}$ such that ${\varvec{B}}={{\,\textrm{curl}\,}}{\varvec{A}}$ and using the gauge condition ${{\,\textrm{div}\,}}{\varvec{A}}=0$.

The heat equation

3 $$\begin{aligned} \rho c \frac{\partial T}{\partial t} + {{\,\textrm{div}\,}}{\varvec{q}} = P, \end{aligned}$$

is obtained from an energy balance, where $\rho c$ is the (non-linear) volumetric heat capacity (VHC), *P* is a source term and the heat flux ${\varvec{q}}$ is expressed through the constitutive law

4 $$\begin{aligned} {\varvec{q}} = - \kappa \text {grad}\, T, \end{aligned}$$

and $\kappa$ denotes the (non-linear) thermal conductivity. At interfaces between different materials, the interface conditions $[T]=0$ and $[{\varvec{n}} \cdot {\varvec{q}}]=0$ apply. The initial condition $T(t=0)=T_0$ is assumed to be known.

The electrical circuit is described by Kirchhoff’s laws

5 $$\begin{aligned} \sum _{k=1}^{N_I} I_k =0, \qquad \sum _{k=1}^{N_V} V_k =0, \end{aligned}$$

which describe the fact that the sum of $N_I$ currents meeting at a node are zero and the sum of $N_V$ voltages in a closed loop are zero. The voltage difference over a resistor being $V= IR$ and over an inductor being $V= -L \frac{\textrm{d}I }{\textrm{d}t}$ and, for a conductor excited by ${\varvec{J}}^{ext}$ the current flowing in this conductor is $I = \int _S {\varvec{J}}^{ext} \cdot {\varvec{n}} \textrm{d}S$ where *S* is the cross-sectional surface area of the conductor. The initial condition $I(t=0)=I_0$ is assumed to be known.

### Superconducting materials

In ordinary conductors, which we denote by $\Omega _c$, the total current which enters at one end of the conductor and leaves at the other, is linearly related to the voltage drop between the two ends. In such conductors, the conduction of electricity leads to the dissipation of energy (known as Ohmic heating) summing to

6 $$\begin{aligned} \int _{\Omega _c} P^{Ohm} \textrm{d}\Omega = \int _{\Omega _c} {\varvec{J}}^o \cdot {\varvec{E}} \textrm{d}\Omega , \end{aligned}$$

which, in turn, is proportional to the conductor’s resistance.

**Fig. 1:**
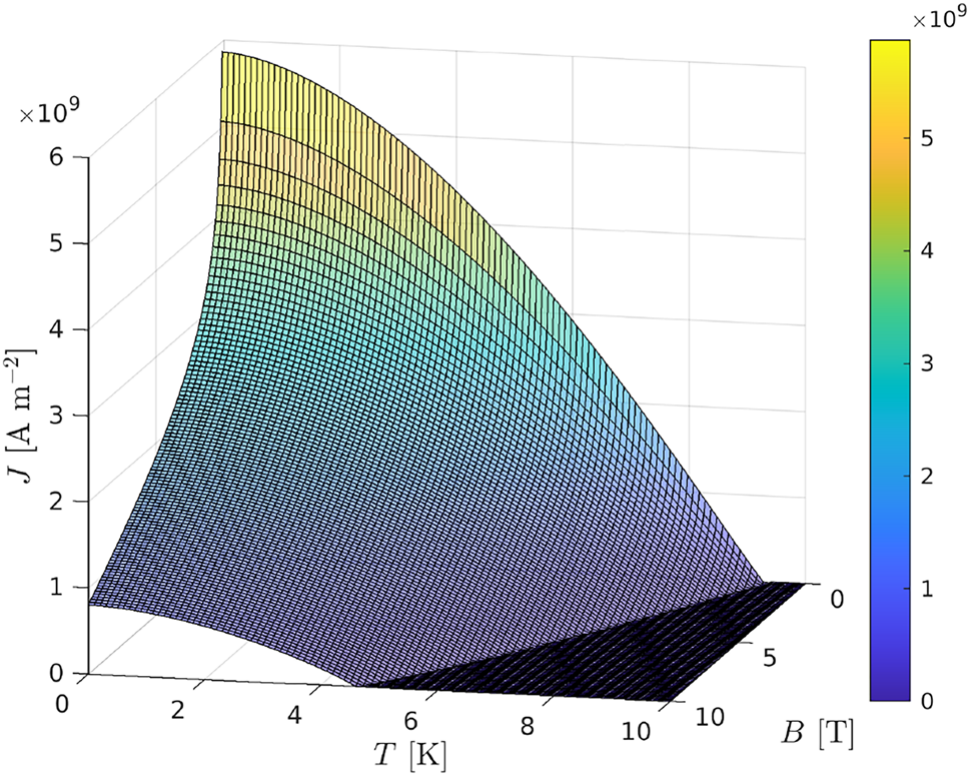
An illustration of the critical surface for NbTi

However, if certain materials are cooled to low temperatures *T* (within a few degrees of absolute zero), for example by being placed in a bath of liquid helium, they conduct electricity without resistance. Such materials are called superconductors. When a current is passed through a superconductor it produces a magnetic field, but this magnetic field does not enter the superconductor. When a material becomes superconducting it expels any magnetic field inside it through a process called the Meisner effect. The Meisner effect is one reason for regarding superconducting materials as magnetic materials. Another reason for regarding superconductors as magnetic materials is that superconductors can become normal conducting if the (tangential) magnetic field exceeds a critical value. Since the magnetic field, in turn, depends on the current this gives rise to so called critical surfaces, which describe the combinations of the triple $(|{\varvec{B}}|, T, I)$ (or equivalently $(|{\varvec{B}}|, T, |{\varvec{J}}^{ext}|)$) for which the material is superconducting (below the critical surface) and normal conducting (above the critical surface). For example, the critical surface for the NbTi material is shown in Fig. 1.

There are several different mathematical models for describing the NbTi critical surface with most following the fit in [6]. The values of these fitting parameters vary between different data sets and we have chosen to use the values provided in [12], which produces the results shown in Fig. 1.

### Magnet coils, cables and wire

**Fig. 2:**
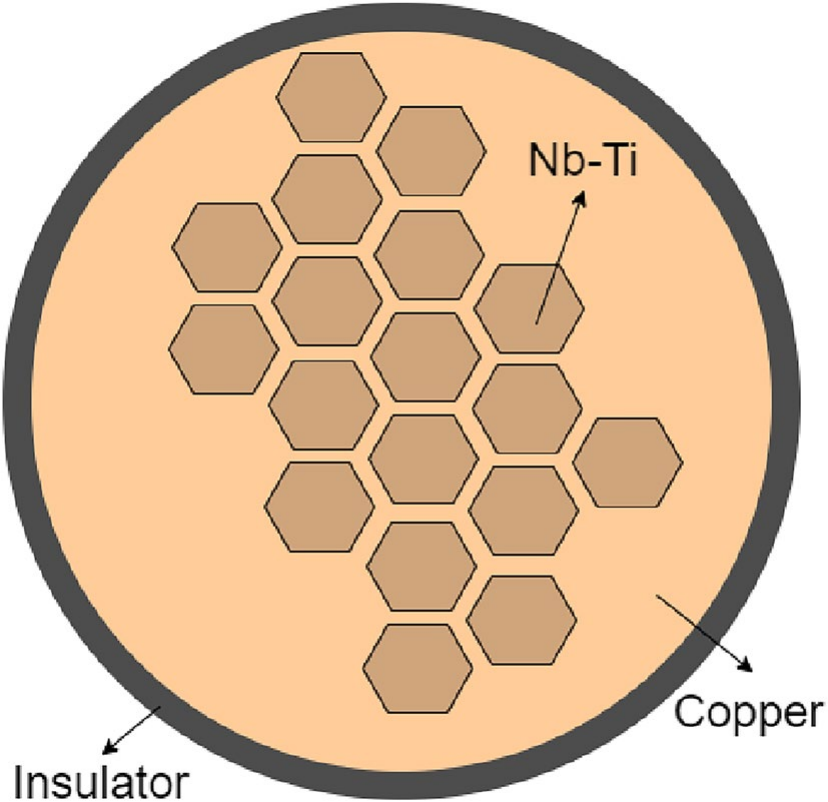
An illustration of the cross section of a typical MRI cable with NbTi filaments, copper filaments and an insulating layer

The main magnet of an MRI scanner is used to produce a strong magnetic field across the bore of the scanner. Superconducting materials are ideal materials for magnets, since once they have been brought upto field, they conduct electricity without resistance. In practice, the main magnet itself is made up of a number of coils with each coil itself being a winding of stranded wire. To ensure a cost effective and safe design, the multiple strands are typically made of NbTi filaments embedded in a copper (Cu) matrix with insulators, as illustrated in Fig. 2, and are bound together. One particular common construction is known as a Rutherford cable [27], which can be used to form the coils of accelerator magnets where extremely high currents are required. We denote the region of the coils as $\Omega _s$.

Each of the materials that form $\Omega _s$ have non-linear constitutive models, which depend on the field quantities and arise from curves that have been fitted to data from experiments. Typical dependence is as follows: For copper, $\rho ^e(T,RRR)$ where *RRR* is the residual resistance ratio and given by $RRR:=\rho ^e(T=273 \text { K})/\rho ^e(T=4 \text { K})$, $\rho c = (\rho c)(| {\varvec{B}}|,T)$ and $\kappa = \kappa (|{\varvec{B}}|,T, RRR)$; For NbTi, $\rho ^e=\rho ^e(T)$, $\rho c = (\rho c)(| {\varvec{B}}|,T)$ and $\kappa =\kappa (T)$ while for insulating materials $\rho c = (\rho c)(| {\varvec{B}}|,T)$ and $\kappa =\kappa (T)$. Mathematical functions describing these constitutive relationships, which have been obtained from curve fitting, along with graphs of the non-linear behaviour of the materials are presented in [21].

The small scale features of the wire strands are too small to be resolved within the computational scheme and instead a homogenised model is typically employed e.g. [5, 11, 21]

7a $$\begin{aligned} \rho ^e({\varvec{x}},t) =&f_{Cu} \rho ^e(T({\varvec{x}},t),RRR)|_{Cu}\nonumber \\&+f_{NbTi} \rho ^e(T({\varvec{x}},t),RRR)|_{NbTi}, \end{aligned}$$

7b $$\begin{aligned} (\rho c )({\varvec{x}},t) =&f_{Cu} (\rho c)( | {\varvec{B}} ( {\varvec{x}},t) |,T({\varvec{x}},t)) |_{Cu}\nonumber \\&+ f_{NbTi} (\rho c)( | {\varvec{B}}({\varvec{x}},t)|,T({\varvec{x}},t)) |_{NbTi} \nonumber \\&+f_{Insul} (\rho c)( | {\varvec{B}}({\varvec{x}},t)|,T({\varvec{x}},t)) |_{Insul} \nonumber \\&+f_{Epoxy} (\rho c)( | {\varvec{B}}({\varvec{x}},t)|,T({\varvec{x}},t)) |_{Epoxy}, \end{aligned}$$

7c $$\begin{aligned} \kappa ({\varvec{x}},t) =&f_{Cu} \kappa (|{\varvec{B}} ( {\varvec{x}},t) |,T ( {\varvec{x}},t) , RRR)|_{Cu}\nonumber \\&+f_{NbTi}\kappa (T ( {\varvec{x}},t) )|_{NbTi} \nonumber \\&+f_{Insul}\kappa (T ( {\varvec{x}},t) )|_{Insul} + f_{Epoxy} \kappa (T ( {\varvec{x}},t) )|_{Epoxy} , \end{aligned}$$

where $f_{Cu}$, $f_{NbTi}$, $f_{Insul}$ and $f_{Epoxy}$ denote the volume fractions of Cu, NbTi, insulating material and epoxy resin, respectively.

As well as homogenising the material properties, it is also important to consider how the circuit *I* is distributed over the coil winding, which is accomplished by the introduction of a winding function ${\varvec{\chi }}$ through

8 $$\begin{aligned} {\varvec{J}}^{ext} = I {\varvec{\chi }}. \end{aligned}$$

While different winding functions are possible [25], for a current flowing in an azimuthal direction and assuming a uniform distribution of the current over the cross-section then ${\varvec{\chi } }:= (N/A_s) {\varvec{e}}_\phi$ where *N* is the number of turns and $A_s$ is the cross-sectional area of the coil is the most commonly used.

If the coil changes from superconducting to normal conducting it will become resistive with resistance given by

9 $$\begin{aligned} R = \int _{\Omega _s} {\varvec{\chi }} \cdot ( \rho ^e {\varvec{\chi }}) \textrm{d}\Omega . \end{aligned}$$

Furthermore, the presence of resistance will lead to a change in current described by Kirchhoff’s laws and this can lead to time–varying electromagnetic fields. This means that the coils can generate eddy currents, which further perturbs the electromagnetic fields. Within the homogenised model, the eddy currents that arise due to the inter-filament coupling are accounted for through the magnetisation term [7]

10 $$\begin{aligned} {\varvec{M}} = - \mu _0^{-1} \tau _{eq} \frac{\partial {\varvec{B}}}{\partial t}, \end{aligned}$$

where $\tau _{eq}$ is an equivalent time constant that depends on the particular coil winding and configuration, which is used to quantify the inter-filament coupling loss [, Chapt 3]. In general, 20$\tau _{eq}$ is a rank-2 tensor that is assumed to be positive definite in $\Omega _s$ and zero elsewhere [7]. A range of different models have been proposed to model this time constant, which depends on the cable parameters (including the magneto-resistivity of the strand’s copper matrix, the filament’s diameter and twist pitch [5]), one possible choice is proposed in [11], [, pg. 26–27] in the form of the positive scalar^20^

11 $$\begin{aligned} \tau _{eq} =\frac{\mu _0}{2} \left( \frac{l_f}{2\pi } \right) ^2 \frac{1}{(c_0 + c_1 | {\varvec{B}} | )f_{NbTi,eff}}, \end{aligned}$$

in $\Omega _s$ where $l_f$ is the filament twist pitch and $c_0$ and $c_1$ are parameters found from experiments [11]. In this expression, $c_0 + c_1 | {\varvec{B}} |$ can recognised as a model for the resistivity of NbTi. In general, $f_{NbTi,eff}$ is one of two possible cases

12 $$\begin{aligned} f_{NbTi,eff} = \frac{1-f_{NbTi}}{1+f_{NbTi}}, \qquad f_{NbTi,eff} = \frac{1+f_{NbTi}}{1-f_{NbTi}}, \end{aligned}$$

with the former expression for low-contact resistance and the latter for high-contact resistance. A more general and alternative expression for (11) is proposed by [31] and their formulation is equivalent to

13 $$\begin{aligned} \tau _{eq} =\frac{\mu _0}{2} \left( \frac{l_f}{2\pi } \right) ^2 \frac{1}{\rho ^e f_{NbTi,eff}}, \end{aligned}$$

where the denominator now includes $\rho ^e$ as the resistivity of the bulk rather than just NbTi. As our interest lies in a superconductor with NbTi filaments that exhibit a high-contact resistance, we use $f_{NbTi,eff} = {(1+f_{NbTi})}/{(1-f_{NbTi})}$ and apply (13). Further refinements are possible by additionally considering inter-strand coupling currents and associated losses [5], however, these require additional information about the coil winding that may not be available.

The coils are immersed in a non-conducting bath $\Omega _{bath}$ of liquid Helium in state of He-I with initial temperature $T_0 =4.2$ K and properties $\rho c =10^5 \text { Jm}^3 \text {K}^{-1}$ and $\kappa =0.02 \text { Wm}^{-1} \text {K}^{-1}$. The non-conducting bath is itself is contained by one or more conducting shields $\Omega _c$, with additional conducting formers also making up part of $\Omega _c$. The shields and formers making up $\Omega _c$ are normal conducting, typically made of aluminium and have conductivity typically ranging $1 \times 10^7 \le \gamma \le 3.5\times 10^7 \text { Sm}^{-1}$ and are non-magnetic with permeability $\mu _0$. The other properties of aluminium are assumed to be $\kappa = 237 \text { Wm}^{-1} \text {K}^{-1}$ and $(\rho c) =2.422\times 10^6 \text { Jm}^3 \text {K}^{-1}$. In the presence of time-varying fields, $\Omega _c$, similar to  $\Omega _s$, can experience eddy currents that can further perturb the results.

**Fig. 3:**
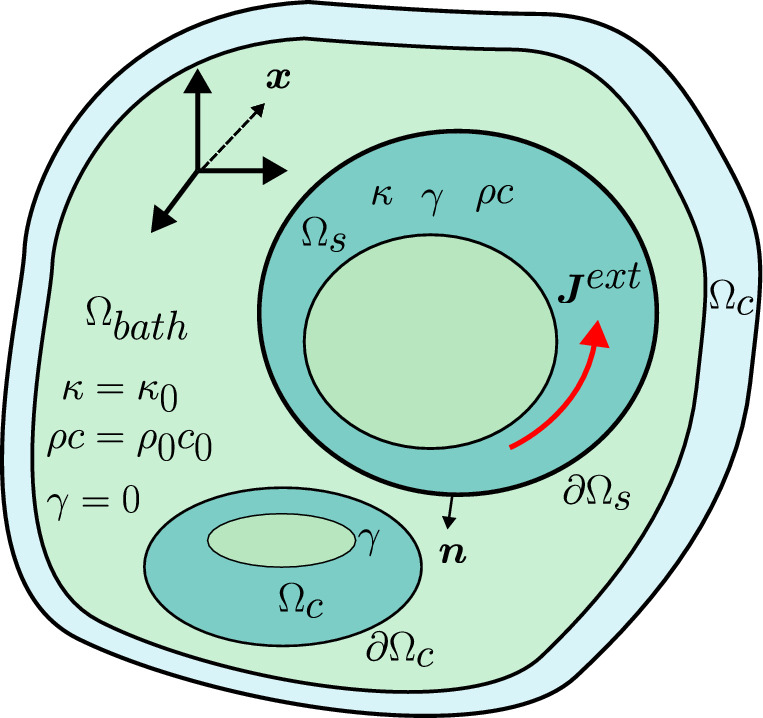
General representation of the stranded coil model, illustrating the stranded coil region $\Omega _s$ with $\gamma$, $\rho c$ and $\kappa$ given by the non-linear models (7), the conducting region $\Omega _c$ with homogeneous isotropic $\gamma$ and the thermal bath $\Omega _{bath}$ with $\gamma = 0$ and homogeneous isotropic $\rho c$ and $\kappa$. An excitation is applied by an external current source $\varvec{J}^{ext}$ inside the coils

Figure 3 illustrates one possible situation with a domain $\Omega =\Omega _s \cup \Omega _c \cup \Omega _{bath}$. As illustrated, $\Omega _c\cap \Omega _s = \emptyset$ so that the stranded and conductor domains are disjoint. We assume throughout that $\partial \Omega _c \cap \partial \Omega _{bath} = \partial \Omega _c$ and $\partial \Omega _s \cap \partial \Omega _{bath} = \partial \Omega _s$ so that $\Omega _c$ has only boundaries with $\Omega _{bath}$ and $\Omega _s$ has only boundaries with $\Omega _{bath}$.

### Losses and the source terms

The stranded coils $\Omega _s$ are assumed to be initially cooled to $T_0=4.2$ K and, according to the critical surface shown in Fig. 1, are in a superconducting state. In this state, they do not experience a dissipation of energy unless a trigger is activated to start the process of a quench. Once a trigger is activated, this starts the irreversible process of a magnet quench where by $\Omega _s$ experiences a rise in temperature leading to the triple $(|{\varvec{B}}({\varvec{x}},t)|, T({\varvec{x}},t), |{\varvec{J}}^{ext}({\varvec{x}},t)|)$ moving above the critical surface and the associated locations ${\varvec{x}} \in \Omega _s$ becoming normal conducting at time *t*. Once parts of the coil become normal conducting, they become resistive and this, in turn, generates further heat. Hence, during quench, and normal conducting operation, the source term is of the form

14 $$\begin{aligned} P=P^{Dyn} +P^{Joule} +P^{Hysteresis}+ P^{Heater} + P^{Trigger}, \end{aligned}$$

for ${\varvec{x}} \in \Omega _s$. In the above, the dynamic heating loss associated with magnetisation is given by

15 $$\begin{aligned} P^{Dyn} := - {\varvec{M}} \cdot \frac{\partial {\varvec{B}}}{\partial t} = \mu _0^{-1} \tau _{eq} \left| \frac{\partial {\varvec{B}} }{\partial t} \right| ^2, \end{aligned}$$

The hysteresis losses [31], defined as

16 $$\begin{aligned} P^{Hysteresis} := \frac{8 a_{sc}N}{3 \pi A_s} \left| |{\varvec{B}}| \frac{\textrm{d}I}{\textrm{d}t} + I \left| \frac{\partial {\varvec{B}}}{\partial t} \right| \right| , \end{aligned}$$

are associated with the magnetisation energy deposited in the superconductor when exposed to a time varying magnetic field. These loss terms are important as they lead to an increase in temperature of neighbouring coils that have not yet quenched. This, in turn, makes the triple $(|{\varvec{B}} |,I,T)$ at locations in these neighbouring coils move above the critical surface causing them to quench. Without including $P^{Hysteresis}$ (or additional triggers $P^{Trigger}$, see below), a neighbouring coil would not be able to quench as there would only be small variations in temperature within the neighbouring coil in the model and the triple $(|{\varvec{B}} |,I,T)$ would remain below the critical surface for positions in this coil.

The Joule heating is defined as

17 $$\begin{aligned} P^{Joule} := \rho ^e \left| {\varvec{J}}^{ext} \right| ^2 = \rho ^e I^2 |{\varvec{\chi }}|^2, \end{aligned}$$

and is associated with the heating effects due to conducting materials and, for superconductors, this only starts to play a role once the conductor has started to transition from the superconducting to normal conducting state.

The additional terms are $P^{Heater}$, which is a source term associated with user prescribed heating of the coil, and $P^{Trigger}$, which is a user prescribed source term describing the trigger of the quench.

Conducting regions (by which we mean massive conductors and not stranded conductors) do not experience dissipation of energy prior to quench as the problem is assumed to be in steady state. Once a quench begins, they experience a dissipation of energy through an Ohmic heating term

18 $$\begin{aligned} P=P^{Ohm} :={\varvec{J}}^o \cdot {\varvec{E}} = \gamma | {\varvec{E}}|^2, \end{aligned}$$

in $\Omega _c$. We assume adiabatic conditions for the helium bath so that $P=0$ in $\Omega _{bath}$, which, together with the properties of Helium, imply that negligible heating of $\Omega _{bath}$ occurs even if $\Omega _s$ and $\Omega _c$ experience heating. This could be refined by including additional source terms.

## Transmission problems

### Initial conditions

To obtain $T_0$, we assume that the magnet is initially in a steady state of superconducting operation where $P=0$ for all regions. This means that $T_0$ is the solution to the steady state problem: Find $T_0 \in {{\mathbb {R}}}$ such that

19a $$\begin{aligned} {{\,\textrm{div}\,}}(\kappa {{\,\textrm{grad}\,}}T_0)&= 0 & \text {in }\Omega , \end{aligned}$$

19b $$\begin{aligned} {[}T_0]&= 0 & \text {on }\partial \Omega _c\cup \partial \Omega _s, \end{aligned}$$

19c $$\begin{aligned} {[}{\varvec{n}} \cdot (\kappa {{\,\textrm{grad}\,}}T_0 )]&= 0 & \text {on }\partial \Omega _c\cup \partial \Omega _s, \end{aligned}$$

19d $$\begin{aligned} T_0&= T_{\partial \Omega } & \text {on }\partial \Omega , \end{aligned}$$

and﻿ the solution to this system (independent of $\kappa$) is $T_0 = T_{\partial \Omega }$ provided that $T_{\partial \Omega }$ is constant.

From the electromagnetic perspective, the following system is solved: Find ${\varvec{A}}_0 \in {{\mathbb {R}}}^3$ such that

20a $$\begin{aligned} \displaystyle {{\,\textrm{curl}\,}}\mu ^{-1} {{\,\textrm{curl}\,}}\varvec{A}_0&= \varvec{0} & \text{ in } \Omega _{c}, \end{aligned}$$

20b $$\begin{aligned} \displaystyle {{\,\textrm{curl}\,}}\mu ^{-1} {{\,\textrm{curl}\,}}\varvec{A}_0&= I_0(t) \varvec{\chi } & \text{ in } \Omega _{s}, \end{aligned}$$

20c $$\begin{aligned} \displaystyle {{\,\textrm{curl}\,}}\mu _0^{-1} {{\,\textrm{curl}\,}}\varvec{A_0}&= \varvec{0} & \text{ in } \Omega _{bath}, \end{aligned}$$

20d $$\begin{aligned} \displaystyle {{\,\textrm{div}\,}}\varvec{A}_0&= 0 & \text{ in } \Omega , \end{aligned}$$

20e $$\begin{aligned} \displaystyle \varvec{n} \times \varvec{A}_0&= \varvec{0} & \text{ on } \partial \Omega , \end{aligned}$$

20f $$\begin{aligned} \left[ \varvec{n} \times \varvec{A}_0\right]&= \varvec{0} & \text{ on } \partial \Omega_c\cup \partial \Omega_s, \end{aligned}$$

20g $$\begin{aligned} \left[ \varvec{n} \times \mu ^{-1} {{\,\textrm{curl}\,}}\varvec{A_0}\right]&= \varvec{0} & \text{ on } \partial \Omega _c \cup \partial \Omega _s , \end{aligned}$$

Note that this will lead to non-zero $|{\varvec{B}}_0 |= |{{\,\textrm{curl}\,}}{\varvec{A}}_0 |$ for ${\varvec{x}} \in \Omega _s$, which is inconsistent with a superconductor that has no ${\varvec{B}}$ field during superconducting operation. However, as $\Omega _s$ is only partly made up of NbTi and also consists of Cu and insulator parts, this is an approximation that we deem to be appropriate and follows as a result of treating this region as homogenised.

### Field-circuit-thermal coupling

Putting together the governing equations and applying the mathematical modelling treatment discussed in Sect. 2, we have the system: Find ${\varvec{A}}\in {{\mathbb {R}}}^3 (0,t_{max}]$, $I(t) \in C^1(0,t_{max}], T\in {{\mathbb {R}}}(0,t_{max}]$ such that

21a $$\begin{aligned}&\displaystyle {{\,\textrm{curl}\,}}\mu ^{-1} {{\,\textrm{curl}\,}}\varvec{A} + \gamma \frac{\partial \varvec{A}}{\partial t} = \varvec{0} \quad \text{ in } \Omega _{c}, \end{aligned}$$

21b $$\begin{aligned}&\displaystyle {{\,\textrm{curl}\,}}\mu ^{-1} {{\,\textrm{curl}\,}}\varvec{A} +{{\,\textrm{curl}\,}}\mu _0^{-1} \tau _{eq} {{\,\textrm{curl}\,}}\frac{\partial \varvec{A}}{\partial t} = I(t) \varvec{\chi } \quad \text{ in } \Omega _{s}, \end{aligned}$$

21c $$\begin{aligned}&\displaystyle {{\,\textrm{curl}\,}}\mu _0^{-1} {{\,\textrm{curl}\,}}\varvec{A} = \varvec{0} \quad \text{ in } \Omega _{bath}, \end{aligned}$$

21d $$\begin{aligned}&\displaystyle {{\,\textrm{div}\,}}\varvec{A} = 0 \quad \text{ in } \Omega _s \cup \Omega _{bath}, \end{aligned}$$

21e $$\begin{aligned}&\displaystyle \varvec{n} \times \varvec{A} = \varvec{0} \quad \text{ on } \partial \Omega , \end{aligned}$$

21f $$\begin{aligned}&\left[ \varvec{n} \times \varvec{A}\right] = \varvec{0} \quad \text{ on } \partial \Omega _c\cup \partial \Omega _s, \end{aligned}$$

21g $$\begin{aligned}&\left[ \varvec{n} \times \mu ^{-1} {{\,\textrm{curl}\,}}\varvec{A}\right] = \varvec{0} \quad \text{ on } \partial \Omega _c, \end{aligned}$$

21h $$\begin{aligned}&\left[ \varvec{n} \times \mu _0^{-1} {{\,\textrm{curl}\,}}\varvec{A}\right] - \varvec{n} \times \mu _0^{-1}\tau _{eq}{{\,\textrm{curl}\,}}\frac{\partial \varvec{A}}{\partial t}\bigg |^{-} = \varvec{0} \quad \text{ on } \partial \Omega _s,\end{aligned}$$

21i $$\begin{aligned}&L \frac{\textrm{d}I}{\textrm{d}t} +IR=- \int _{\Omega _s} \varvec{\chi } \cdot \frac{\partial \varvec{A}}{\partial t}\textrm{d}\Omega +IR= 0 \quad \end{aligned}$$

21j $$\begin{aligned}&\rho c \frac{\partial T}{\partial t} - {{\,\textrm{div}\,}}(\kappa {{\,\textrm{grad}\,}}T) -\gamma \left| \frac{\partial \varvec{A}}{\partial t} \right| ^2 =0 \quad \text{ in } \Omega _{c}, \end{aligned}$$

21k $$\begin{aligned}&\rho c \frac{\partial T}{\partial t} - {{\,\textrm{div}\,}}(\kappa {{\,\textrm{grad}\,}}T) - P^{Trigger} -P^{Heater} \quad \nonumber \\&-\frac{8 a_{sc}N}{3 \pi A_s} \left| |{{\,\textrm{curl}\,}}{\varvec{A}}| \frac{\textrm{d}I}{\textrm{d}t} + I \left| \frac{\partial {{\,\textrm{curl}\,}}{\varvec{A}} }{\partial t} \right| \right|\nonumber \\& - \mu _0 ^{-1} {\tau }_{eq} \left| {{\,\textrm{curl}\,}}\frac{\partial {\varvec{A}}}{\partial t} \right| ^2 -\rho ^e I^2 |{\varvec{\chi }}|^2 =0 \quad \text{ in } \Omega _{s}, \end{aligned}$$

21l $$\begin{aligned}&\rho c \frac{\partial T}{\partial t} - {{\,\textrm{div}\,}}(\kappa {{\,\textrm{grad}\,}}T) = 0 \quad \text{ in } \Omega _{bath}, \end{aligned}$$

21m $$\begin{aligned}& [T] = 0, \qquad[{\varvec{n}} \cdot (\kappa {{\,\textrm{grad}\,}}T )] = 0\quad \text {on }\partial \Omega _c\cup \partial \Omega _s,\end{aligned}$$

21n $$\begin{aligned}&T = T_{\partial \Omega } \quad \text {on }\partial \Omega ,\end{aligned}$$

21o $$\begin{aligned}&\varvec{A}(t = 0) = \varvec{A}_0 \quad \text {in }\Omega , \end{aligned}$$

21p $$\begin{aligned}&I(t=0) = I_0 , \end{aligned}$$

21q $$\begin{aligned}&T(t=0) = T_0\quad \text {in }\Omega . \end{aligned}$$

Note that the above (21i) is presented for the situation of a single coil model, but can also be extended to multiple coils. Also note that the choice of gauge in (21d) is appropriate given our choice of winding function, but may need to be changed for a different choice of ${\varvec{\chi }}$. Our interest is in rotationally symmetric MRI coils where $\Omega$ can be described as $\Omega =\{ (r,\phi ,z): (r,z) \in \Omega ^m, \phi \in [0,2\pi )\}$, with $\Omega ^m$ being a rotationally invariant meridian plane, ${\varvec{A}} = A_\phi (r,z) {\varvec{e}}_\phi$ and $T= T(r,z)$ independent of $\phi$ and in this case (21d) is automatically satisfied. While it is possible to apply these simplifications to (21), it is more instructive to first obtain the weak formulation of the three-dimensional problem as this will lead to a more natural treatment and allows us to circumvent the 1/*r* singularities associated with the radial axis that would otherwise plague the finite element approximation of the weak form in the axisymmetric setting. Specifically, in the following, we set out the weak treatment of the initial conditions in three-dimensions and explain their reduction to the axisymmetric setting. We follow a similar treatment of the field-circuit-thermal coupling and also include details of the temporal, non-linear and spatial discretisation We also additionally remark on the necessary extensions needed for a fully three-dimensional computational treatment.

## Continuous weak forms

### Initial conditions

The weak form for the initial temperature field is: Find $T_0 \in V(T_{\partial \Omega })$ such that

22 $$\begin{aligned} \int _\Omega \kappa {{\,\textrm{grad}\,}}T_0 \cdot {{\,\textrm{grad}\,}}\delta T \textrm{d}\Omega =0 , \end{aligned}$$

for all $\delta T \in V(0)$ where

$$\begin{aligned} V(T_{\partial \Omega } ) := \{ T \in H^1( \Omega ) : T = T_{\partial \Omega }\text { on }\partial \Omega \} . \end{aligned}$$

The solution to this system (independent of $\kappa$) is $T_0 = T_{\partial \Omega }$ provided that $T_{\partial \Omega }$ is constant. By writing $\textrm{d}\Omega = r\textrm{d}r \textrm{d}z \textrm{d}\phi$, constructing a geometry $\Omega ^m$ that is independent of $\phi$, and for problems where $T_0$ is independent of $\phi$, the corresponding axisymmetric problem, when expressed in terms of a bilinear form, is: Find $T_0 \in V_a (T_{\partial \Omega })$ such that

23 $$\begin{aligned} A_{\Omega ^m}(T_0, \delta T) : = \int _{\Omega ^m} \kappa {{\,\textrm{grad}\,}}_m T_0 \cdot {{\,\textrm{grad}\,}}_m \delta T r \textrm{d}r \textrm{d}z =0, \end{aligned}$$

for all $\delta T \in V_a(0)$, where ${{\,\textrm{grad}\,}}_m T:=( \partial T/\partial r) {\varvec{e}}_r + (\partial T/\partial z) {\varvec{e}}_z$ and

$$\begin{aligned} V_a (T_{\partial \Omega } ) := \{ T \in H^1( \Omega ^m ) : T = T_{\partial \Omega }\text { on }\partial \Omega ^m \setminus \{r=0\} \} . \end{aligned}$$

The corresponding weak form for the initial vector potential is: Find $\varvec{A}_0 \in Z$ such that

24 $$\begin{aligned} \int _{\Omega } \mu ^{-1} {{\,\textrm{curl}\,}}\varvec{A}_0 \cdot {{\,\textrm{curl}\,}}\delta \varvec{A}_0 \textrm{d}\Omega = I_0 \int _{\Omega _s} \varvec{\chi } \cdot \delta \varvec{A}_0 \textrm{d}\Omega , \end{aligned}$$

for all $\delta {\varvec{A}}_0 \in Z$, where

$$\begin{aligned} Z := \{ {\varvec{A}} \in {\varvec{H}} (\hbox {curl} ) : {{\,\textrm{div}\,}}{\varvec{A}} = 0 \text { in }\Omega , {\varvec{n}} \times {\varvec{A}} = {\varvec{0}} \text { on }\partial \Omega \} . \end{aligned}$$

Following the approach in [14] for axisymmetric problems, this reduces to: Find ${\hat{A}}_{\phi ,0} \in V_a(0)$ such that

25 $$\begin{aligned} B_{\Omega ^m} ( {\hat{A}}_{\phi ,0}, \delta {\hat{A}}_\phi ) = f _{\Omega _s^m}( \delta {\hat{A}}_\phi ; I ), \end{aligned}$$

for all $\delta {\hat{A}}_\phi \in V_a(0)$ and is expressed in terms of the bilinear and linear forms

$$\begin{aligned} B_{\Omega ^m} ( u, v):&= \int _{\Omega ^m} \frac{\mu ^{-1}}{r} {{\,\textrm{grad}\,}}_m (r^2 u ) \cdot {{\,\textrm{grad}\,}}_m (r^2 v )\textrm{d}r \textrm{d}z, \\ f_{\Omega ^m}( v ; I ):&= \frac{IN}{A_s} \int _{\Omega _s^m} v r^2 \textrm{d}r \textrm{d}z , \end{aligned}$$

for place holder fields *u* and *v*. In the above, $A_{\phi ,0} = r {\hat{A}}_{\phi ,0}$ has been introduced to avoid having to solve for fields in weighted spaces and to resolve the 1/*r* singularities associated with $r\rightarrow 0$.

### Field-circuit-thermal coupling

Given a set of suitable trial weak solutions $(\varvec{A}(t),I(t),T(t)) \in (X \times C^1 \times V(T_{\partial \Omega }))$, the weak residual equations and associated initial conditions can be established as

26a $$\begin{aligned} R_A (\delta {\varvec{A}}; {\varvec{A}},I)&:= \int _{\Omega } \mu ^{-1} {{\,\textrm{curl}\,}}\varvec{A} \cdot {{\,\textrm{curl}\,}}\delta \varvec{A} \textrm{d}\Omega \nonumber \\&\quad +\int _{\Omega _s} \mu _0^{-1} \tau _{eq} {{\,\textrm{curl}\,}}\frac{\partial {\varvec{A}}}{\partial t} \cdot {{\,\textrm{curl}\,}}\delta {\varvec{A}} \textrm{d}\Omega \nonumber \\&\quad + \int _{\Omega _c} \gamma \frac{\partial \varvec{A}}{\partial t} \cdot \delta \varvec{A} \textrm{d}\Omega - I \int _{\Omega _s} \varvec{\chi } \cdot \delta \varvec{A} \textrm{d}\Omega , \end{aligned}$$

26b $$\begin{aligned} R_I({\varvec{A}},I)&:= - \int _{\Omega _s} {\varvec{\chi }}\cdot \frac{\partial {\varvec{A}}}{\partial t} \textrm{d}\Omega +IR, \end{aligned}$$

26c $$\begin{aligned} R_T(\delta T; \varvec{A},T,I)&:= \int _{\Omega } \rho c \frac{\partial T}{\partial t} \delta T \textrm{d}\Omega + \int _{\Omega } \kappa {{\,\textrm{grad}\,}}T \cdot {{\,\textrm{grad}\,}}\delta T \textrm{d}\Omega \nonumber \\&\quad - \int _{\Omega _s}\mu _0^{-1} \tau _{eq} \left| \frac{\partial ({{\,\textrm{curl}\,}}\varvec{A})}{\partial t}\right| ^2 \delta T \textrm{d}\Omega \nonumber \\&\quad -\int _{\Omega _s} \frac{8 a_{sc}N}{3 \pi A_s} \left| |{{\,\textrm{curl}\,}}{\varvec{A}}| \frac{\textrm{d}I}{\textrm{d}t} + I \left| \frac{\partial {{\,\textrm{curl}\,}}{\varvec{A}} }{\partial t} \right| \right| \delta T \textrm{d}\Omega \nonumber \\ &\quad - \int _{\Omega _c} \gamma \left| \frac{\partial \varvec{A}}{\partial t}\right| ^2 \delta T \textrm{d}\Omega \nonumber \\&\quad -\frac{I^2N^2}{A_s^2} \int _{\Omega _s} \rho ^e \delta T \textrm{d}\Omega - \int _{\Omega _s} (P^{Trigger} + P^{Heater}) \delta T \textrm{d}\Omega ,\end{aligned}$$

26e $$\begin{aligned} {\varvec{A}}(t=0)&= {\varvec{A}}_0 \ \text {in }\Omega , \end{aligned}$$

26f $$\begin{aligned} I(t=0)&= I_0 , \end{aligned}$$

26g $$\begin{aligned} {T}(t=0)&= T_0 \ \text {in }\Omega , \end{aligned}$$

for all $\delta \varvec{A} \in X$, $\delta T \in V(0)$ where

$$\begin{aligned} X := \{ {\varvec{A}} \in {\varvec{H}} (\hbox {curl} ) : {{\,\textrm{div}\,}}{\varvec{A}} = 0 \text { in }\Omega _s \cup \Omega _{bath}, {\varvec{n}} \times {\varvec{A}} = {\varvec{0}} \text { on }\partial \Omega \} . \end{aligned}$$

In an axisymmetric setting, given a set of trial solutions $({\hat{A}}_\phi (t),I(t),T(t)) \in (V_a(0) \times C^1 \times V_a(T_{\partial \Omega }))$, this reduces to

27a $$\begin{aligned} R_A \left( \delta {\hat{A}}_\phi ; {\hat{A}}_\phi ,I\right)&= B_{\Omega ^m} \left( {\hat{A}}_{\phi } , \delta {\hat{A}}_\phi \right) \nonumber \\&+ B_{\Omega _s^m} \left( \partial {\hat{A}}_{\phi } / \partial t, \delta {\hat{A}}_\phi \right) \nonumber \\&+ C_{\Omega _c^m}\left( \partial {\hat{A}}_{\phi }/ \partial t , \delta {\hat{A}}_\phi \right) - f_{\Omega _s^m} \left( \delta {\hat{A}}_\phi ; I\right) , \end{aligned}$$

27b $$\begin{aligned} R_I ({\hat{A}}_\phi ,I )&= - f_{\Omega _s^m} \left( \partial {\hat{A}}_\phi /\partial t; 1\right) + IR , \end{aligned}$$

27c $$\begin{aligned} R_T\left( \delta T; {\hat{A}}_\phi , T, I\right)&= D_{\Omega ^m} \left( \partial T/\partial t, \delta T\right) \nonumber \\&+ A_{\Omega ^m}(T,\delta T) - g_{\Omega _s^m} \left( \delta T ; I, {\hat{A}}_\phi , \partial {\hat{A}}_\phi / \partial t\right) , \end{aligned}$$

27d $$\begin{aligned} {\hat{A}}_\phi (t=0)&= {\hat{A}}_{\phi , 0} \ \text {in }\Omega , \end{aligned}$$

27e $$\begin{aligned} I(t=0)&= I_0 , \end{aligned}$$

27f $$\begin{aligned} T(t=0)&= T_0 \ \text {in }\Omega , \end{aligned}$$

for all $\delta {\hat{A}}_\phi \in V_a (0)$ and $\delta T \in V_a(0)$ where the additional bilinear and linear forms needed are

$$\begin{aligned} B_{\Omega _s^m} (u , v ) : =&\int _{\Omega _s^m} \frac{\mu _0^{-1}\tau _{eq}}{r} {{\,\textrm{grad}\,}}_m \left( r^2 u \right) \cdot {{\,\textrm{grad}\,}}_m \left( r^2 v \right) \textrm{d}r \textrm{d}z, \\ C_{\Omega _c^m}( u , v ) :=&\int _{\Omega _c^m} \gamma u v r^3 \textrm{d}r \textrm{d}z , \\ D_{\Omega ^m}(u ,v ) : =&\int _{\Omega ^m} \rho c u v r \textrm{d}r \textrm{d}z, \\ g_{\Omega _s^m} \left( v ; I, {\hat{A}}_\phi , \partial {\hat{A}}_\phi / \partial t \right) :=&\int _{\Omega _s^m } \frac{8 a_{sc}N}{3 \pi A_s} \left| |{{\,\textrm{curl}\,}}{\varvec{A}}| \frac{\textrm{d}I}{\textrm{d}t}\right. \\&\left. + I \left| \frac{\partial {{\,\textrm{curl}\,}}{\varvec{A}} }{\partial t} \right| \right| v r \textrm{d}r \textrm{d}z \\&+ \int _{\Omega _c^m } \gamma \left| \frac{\partial \varvec{A}}{\partial t}\right| ^2 v r \textrm{d}r \textrm{d}z\\&+\frac{I^2N^2}{A_s^2} \int _{\Omega _s^m } \rho ^e v r \textrm{d}r \textrm{d}z \\&+ \int _{\Omega _s^m } \left( P^{Trigger} + P^{Heater}\right) v r\textrm{d}r \textrm{d}z, \end{aligned}$$

and we recall that ${{\,\textrm{curl}\,}}{\varvec{A}}$ and $\frac{\partial \varvec{A}}{\partial t}$ can be expressed in terms of ${\varvec{A}} = A_\phi {\varvec{e}}_\phi = r {\hat{A}}_\phi {\varvec{e}}_\phi$.

## Discrete weak forms

### Discrete initial conditions

Introducing a finite element partition of $\Omega ^m$, consisting of non-overlapping elements of variable size *h* and order *p* elements, the discrete approximation to (23) is: Find $T_0^{hp} \in W^{hp} \cap V_a (T_{\partial \Omega })$ such that

28 $$\begin{aligned} A_{\Omega ^m}\left( T_0^{hp}, \delta T^{hp}\right) =0, \end{aligned}$$

for all $\delta T^{hp} \in W^{hp} \cap V_a(0)$, where $W^{hp} \subset H^1$ is an appropriate set of $H^1$ conforming finite element shape functions. In a similar way, the discrete approximation to (25) is: Find ${\hat{A}}_{\phi ,0}^{hp} \in W^{hp} \cap V_a(0)$ such that

29 $$\begin{aligned} B_{\Omega ^m} \left( {\hat{A}}_{\phi ,0}^{hp}, \delta {\hat{A}}_\phi ^{hp}\right) = f _{\Omega ^m}\left( \delta {\hat{A}}_\phi ^{hp}; I\right) , \end{aligned}$$

for all $$\delta {\hat{A}}_{\phi }^{hp} \in W^{hp} \cap V_a(0)$$.

### Discrete field-circuit-thermal coupling

We choose to first discretise in time, then present an algorithm for resolving the non-linearity and finally discretise in space. For the temporal discretisation, we choose to employ an un-conditionally stable Euler implicit time integration scheme. This is motivated by the fact that when quench propagates over the coil it does so like shock with a steep temporal gradient. Higher order temporal integration would be well suited to the integration of fields that are smooth with *t* and, while this is the case once the coil has fully quenched, we choose to use the same temporal integration throughout for simplicity. In the following, we drop the subscript $\phi$ on ${\hat{A}}_\phi$ for simplicity of presentation. This leads to the residual equations at time level $\ell$

30a $$\begin{aligned}&R_A \left( \delta {\hat{A}} , {\hat{A}}_{\ell -1} ; {\hat{A}}_\ell ,I_\ell\right) = B_{\Omega ^m} \left( {\hat{A}}_{\ell } , \delta {\hat{A}} \right) \nonumber \\&+ B_{\Omega _s^m} \left( ({\hat{A}}_{\ell }- {\hat{A}}_{\ell -1}\right) /\Delta t , \delta {\hat{A}} ) + C_{\Omega _c^m}\left( \left( {\hat{A}}_{\ell }- {\hat{A}}_{\ell -1}\right) /\Delta t , \delta {\hat{A}}\right) \nonumber \\&- f_{\Omega _s^m} ( \delta {\hat{A}}; I_\ell) , \end{aligned}$$

30b $$\begin{aligned}&R_I ({\hat{A}}_{\ell -1}; {\hat{A}}_\ell ,I_\ell ) = - f_{\Omega _s^m} ( ( {\hat{A}}_\ell - {\hat{A}}_{\ell -1 } ) /\Delta t; 1) + I_\ell R , \end{aligned}$$

30c $$\begin{aligned}&R_T( \delta T, {\hat{A}}_{\ell -1}, T_{\ell -1}; {\hat{A}}_\ell , T_\ell , I_\ell )\nonumber \\&= D_{\Omega ^m} ((T_\ell - T_{\ell -1})/ \Delta t, \delta T) + A_{\Omega ^m}(T_\ell ,\delta T) - \nonumber \\&g_{\Omega _s^m} ( \delta T ; I_\ell , {\hat{A}}_\ell , ({\hat{A}}_\ell - {\hat{A}}_{\ell -1})/\Delta t ) \end{aligned}$$

where $\Delta t:= t_{\ell +1} - t_\ell$ and, for the solution of the coupled system, the fixed point strategy stated in Algorithm 1 is proposed in which the subscripts in square brackets denote the fixed point iterations. The philosophy of this scheme is that it contains two nested fixed point iterations with the inner iteration being focused on solving the coupled system of electromagnetic and thermal equations for a fixed current and then the outer iteration updating the circuit equation and solution for the current. Instead of the proposed fixed point strategy, an alternative would be to apply a consistent linearisation of (30) and apply a Newton–Raphson approach. Given an admissible initial guess, both the fixed point scheme and Newton–Raphson scheme will converge to the same solution [4]. However, although the norm of the residual of the Newton–Raphson algorithm converges quadratically to zero, and the fixed point offers only linear convergence, the initial guess of the former must be much closer to the solution to ensure reliable convergence of the scheme (which may be harder to ensure in practice without smaller timesteps). Furthermore, the application of the Newton–Raphson algorithm would result in a large monolithic solve at each iteration for the coupled system, rather than the smaller linear systems that must be solved for each individual physics in the proposed approach. This could have considerable resource implications, particularly if a fully three-dimensional treatment is considered (see Sect. 5.4). Moreover, in the presented axisymmetric simulations, apart from the time corresponding to when the coil becomes fully quenched, the gradient of the linear convergence of the residual equations is steep and the scheme in Algorithm 1 converges to a small tolerance within only a small number of iterations and, thus, the additional complexity of the Newton–Raphson solver was deemed un-necessary. We provide an example of the convergence behaviour in Sect. 6.2.1.

**Algorithm 1:**
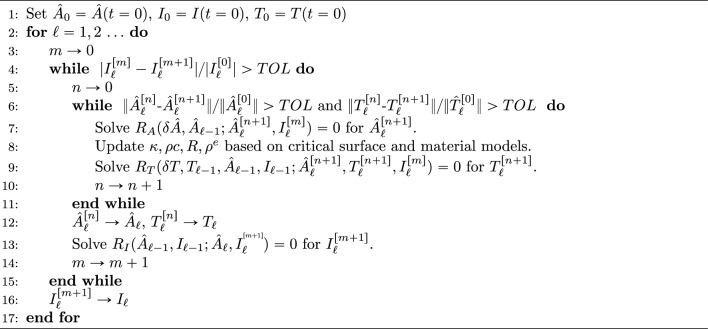
Fixed point algorithm

The spatial discretisation is similar to (28) and (29), given converged discrete solutions ${\hat{A}}_{\ell -1}^{hp}\in W^{hp} \cap V_a(0)$ and $T_{\ell -1}^{hp} \in W^{hp} \cap V_a (T_{\partial \Omega })$ from previous time steps, we proceed as in Algorithm 1 in a staggered manner and first find ${\hat{A}}_\ell ^{[n+1],hp} \in W^{hp} \cap V_a(0)$ such that

31 $$\begin{aligned} R_A \left( \delta {\hat{A}}^{hp}, {\hat{A}}_{\ell -1}^{hp} ; {\hat{A}}_{\ell } ^{[n+1],hp},I_{\ell } ^{[m]} \right) =0, \end{aligned}$$

for all $\delta {\hat{A}}^{hp} \in W^{hp} \cap V_a(0)$ and then we find $T_\ell ^{[n+1],hp} \in W^{hp} \cap V_a (T_{\partial \Omega })$ such that

32 $$\begin{aligned} R_T\left( \delta T^{hp}, T_{\ell -1}^{hp},{\hat{A}}_{\ell -1}^{hp} , I_{\ell -1} ; {\hat{A}}_\ell ^{[n+1],hp},T_\ell ^{[n+1],hp}, I_\ell ^{[m]} \right) =0, \end{aligned}$$

for all $$\delta T ^{hp} \in W^{hp} \cap V_a (0)$$.

### Computational implementation of the axisymmetric problem

We have developed a python code, which is used to interface with the NGSolve finite element library [22–24] for performing the underlying finite element computations to implement Algorithm 1. This includes a user-defined problem file to define the geometry, sub-domains, boundary conditions, mesh spacings, element order *p*, timestep size $\Delta t$ and problem flags for each individual simulation, which uses the NGSolve geometry primitives, multiple regions, to tag boundaries and to tag those regions identified for boundary layers. The Netgen mesh generator is then called to generate an unstructured triangular partition of $\Omega ^m$ where we choose to introduce quadrilateral layers, as defined in the user-defined settings to model high field gradients at material interfaces.

Once the mesh is established, order *p* NGSolve $H^1$ conforming finite element spaces are defined corresponding to $W^{hp} \cap V_a (T_{\partial \Omega })$ and $W^{hp} \cap V_a (0)$, in addition a discrete variant of the $L_2$ conforming finite element space is defined. NGSolve GridFunctions are introduced for the $H^1$ conforming approximations ${\hat{A}}_{\ell }^{[n],hp}$ and $T_\ell ^{[n],hp}$ while an $L_2$ conforming approximation and appropriate GridFunctions are used to represent the material properties $\kappa$, $\rho c$ and $\rho ^e$. These GridFunctions belonging to the aforementioned discrete finite element spaces and are used to represent the discrete approximation and, in particular, the solution coefficients for each field. In an element *e*,

$$\begin{aligned} {\hat{A}}_{\ell }^{[n],hp}|_e&= \sum _{m=1}^M {\hat{A}}_{\ell ,m}^{[n],e} {\vartheta }_m, \\ T_\ell ^{[n],hp} |_e&= \sum _{m=1}^M T_{\ell ,m}^{[n],e} {\vartheta }_m, \\ \kappa ^{hp}|_e&= \sum _{o=1}^O \kappa _{o}^{e} {\psi }_o,\\ (\rho c)^{hp}|_e&= \sum _{o=1}^O (\rho c)_{o}^{e} {\psi }_o, \\ (\rho ^e)^{hp}|_e&= \sum _{o=1}^O (\rho ^e)_{o}^{e} {\psi }_o. \end{aligned}$$

In addition, *M* and *O* denote the number of $H^1$ and $L_2$ conforming functions in the element, which depend on the order *p*, ${\vartheta }_m$ and $\psi _o$ are typical basis functions for the respective spaces and ${\hat{A}}_{\ell ,m}^{[n],e}$, $T_{\ell ,m}^{[n],e}$, $\kappa _{o}^{e}$, $(\rho c)_{o}^{e}$ and $(\rho ^e)_{o}^{e}$ are the solutions coefficients in this element. Note that NGSolve employs the compatible sets of $H^1$ and $L_2$ conforming hierarchic basis functions proposed by Schöberl and Zaglmayr [24, 32].

Prior to the main time integration loop, in Line 1, the discrete approximations ${\hat{A}}_{0}^{hp}\in W^{hp} \cap V_a(0)$ (again dropping subscript $\phi$) and $T_0^{hp}\in W^{hp} \cap V_a(T_{\partial \Omega })$ to the initial conditions are found by solving finite element problems (28) and (29) by specifying the bilinear forms $A_{\Omega ^m}$ and $B_{\Omega ^m}$ and the linear form $f_{\Omega ^m}$, assembling the linear system and solve the system directly for the solution coefficients using similar steps to those setout in the NGSolve documentation. To accelerate these computations, the NGSolve shared memory parallelisation is employed using its built in TaskManager. This generates threads that collaborate on the code block that follows and the work intensive NGSolve commands are internally parallelised.

The next step is the main time integration loop, which begins on line Line 2. At each time step, a fixed-point approach is used to resolve the non-linearity. This is coded through the set of two nested of two while loops where the outer loop is used to converge the circuit model and the inner loop the field equations. We solve (31) for ${\hat{A}}_{\ell } ^{[n+1],hp} \in W^{hp} \cap V_a(0)$ and then (32) for $T_\ell ^{[n+1],hp} \in W^{hp} \cap V_a(T_{\partial \Omega })$. To do this, the bilinear forms $B_{\Omega _s^m}$, $C_{\Omega _c^m}$ and $D_{\Omega ^m}$ and the linear form $g_{\Omega _s^m}$ are additionally specified, the linear system is assembled and the system solved directly for the associated solution coefficients using similar steps to those setout in the NGSolve documentation and again accelerated using the TaskManager.

Since $B_{\Omega _s^m}$ depends on $\tau _{eq}$, $A_{\Omega ^m}$ on $\kappa$, $D_{\Omega ^m}$ on $(\rho c)$ and $g_{\Omega _s^m}$ on $\rho ^e$ further complexities arise. The material variables are replaced by their discrete counterparts $\kappa ^{hp}$, $(\rho c)^{hp}$ and $(\rho ^e)^{hp}$ and their values are set according to the constitutive laws. However, as these laws are highly non-linear (with the relationship either provided through data points or functions that have been previously fitted to data [21][p.g 703-714]), they are unsuitable for directly employing within the finite element computations, and, instead, they are first interpolated using a B-spline function, which is evaluated at the desired integration point locations for computing the finite element local matrices. This is achieved using the NGSolve BSpline CoefficientFunction and then the values of the GridFunctions functions are set. An investigation of the effects of order and the knot-vector on the accuracy of the interpolant (to the available data/fitting function) is undertaken in thesis by one of the authors [17] and these have been chosen so that their relative accuracy is $10^{-3}$. Still further, the form of the constitutive laws depends on whether the position in the stranded conductor is normal or super conducting. To check this, the triple $(| {\varvec{B}}_\ell ^{[n]} |, T_\ell ^{[n]}, |({\varvec{J}}^{ext} )_\ell ^{[n]}|)$ is evaluated to determine whether the coil has become normal conducting and the form of the constitutive law is updated accordingly.

### Extension of the implementation to the three-dimensional case

To allow the treatment of more complex three-dimensional geometries, the approach set out in Sect. 5.3 could be extended to the full three-dimensional case by instead using the three-dimensional weak forms of the problem, written in terms of appropriate bilinear and linear forms. A non-overlapping partition of $\Omega$ (rather than $\Omega ^m$) would be required and unstructured tetrahedral elements would be envisaged due to the availability of automatic unstructured mesh generators for generating the grid around complex geometrical configurations. Rather than thin layers of quadrilaterals, prismatic layers can be used to resolve the high field gradients at material interfaces [10]. Additionally, rather than solve for ${\hat{A}}_{\ell }^{[n+1],hp} \in W^{hp} \cap V_a(0)$, a solution should be sought for ${\varvec{A}}_\ell ^{[n+1],hp}\in X^{hp} \cap X$, where $X^{hp} \subset {\varvec{H}}(\hbox {curl})$ are an appropriate set of ${\varvec{H}}(\hbox {curl})$ conforming basis functions, and for $T_\ell ^{[n],hp} \in W^{hp} \cap V(T_{\partial \Omega })$. While suitable NGSolve GridFunctions are available, and could be used for this purpose, an additional difficulty arises due to the Coulomb gauge conditions that appears in the definitions of *Z* and *X*, which are automatically satisfied in the axisymmetric setting. One remedy to overcome this is to circumvent these gauge condition by regularisation [16, 32], which, for example, would mean that the weak form provided in (24) should be replaced by: Find ${\varvec{A}}_0^\varepsilon \in Z^\epsilon$

33 $$\begin{aligned} \int _{\Omega } \mu ^{-1} {{\,\textrm{curl}\,}}\varvec{A}_0^\varepsilon \cdot {{\,\textrm{curl}\,}}\delta \varvec{A}_0^\varepsilon \textrm{d}\Omega + \varepsilon \int _\Omega \varvec{A}_0^\varepsilon \cdot \delta \varvec{A}_0^\varepsilon \textrm{d}\Omega = I_0 \int _{\Omega _s} \varvec{\chi } \cdot \delta \varvec{A}_0 ^\varepsilon \textrm{d}\Omega , \end{aligned}$$

for all $\delta {\varvec{A}}_0^\varepsilon \in Z^\varepsilon$, where $\varepsilon$ is a small regularisation parameter and

$$\begin{aligned} Z^\varepsilon := \{ {\varvec{A}}^\varepsilon \in {\varvec{H}} (\hbox {curl} ) : {\varvec{n}} \times {\varvec{A}}^\varepsilon = {\varvec{0}} \text { on }\partial \Omega \} . \end{aligned}$$

A similar treatment could also be applied to the residual equation in (26a). Furthermore, compared to the axisymmetric setting, in three-dimensions, an efficient solution of the linear systems resulting from the discretisation of the weak forms is essential and direct solution approaches are expected to be prohibitively expensive due to the high memory requirements. Instead, following the fixed point strategy, the NGSolve finite element library would allow the built-in iterative solvers and preconditioning techniques to be applied, which would not be possible if a monolithic solution approach using Newton–Raphson was applied. As before, $L_2$ conforming approximation and appropriate GridFunctions are used to represent the material properties $\kappa$, $\rho c$ and $\rho ^e$ and the fixed point algorithm specified in Algorithm 1 can easily be adapted and the steps would remain similar.

## Numerical examples

Before considering the application of Algorithm 1 to the quench problem, we first consider the validation of each of the individual physics in Sect. 6.1 and we use these examples to motivate whether *h-*, *p-* or *hp*-refinement is the best strategy for different classes of problems. In particular, in Sect. 6.1.1, we present results for a spherical thermal benchmark problem with a smooth solution. Then, in Sect. 6.1.2 we present time harmonic results for a conducting sphere in a uniform amplitude magnetic field, which has a solution with a steep field gradient due to a material interface. Then, in Sect. 6.2 we show a range of challenging quench simulations, which, while more challenging, has both regions where the field solutions are smooth and have steep field gradients. This includes a single coil benchmark problem in Sect. 6.2.1 and a two coil problem with different configurations in Sects.  6.2.2, 6.2.3 and 6.2.4.

### Single physics validation

The following relative error measures will be used to investigate the convergence behaviour of the single physics benchmark problems

34a $$\begin{aligned}&\Vert e(u) \Vert _{L_2(\Omega ^m)} = \left( \int _{\Omega ^m} |e(u)|^2 r \textrm{d}r \textrm{d}z \Bigg / \int _{\Omega ^m} |u^{exact} |^2 r \textrm{d}r \textrm{d}z \right) ^{1/2} , \end{aligned}$$

34b $$\begin{aligned}\Vert e(u) \Vert _{H^1(\Omega ^m)} &= \left( \int _{\Omega ^m} \left( | e(u)|^2 + | {{\,\textrm{grad}\,}}e(u)|^2\right) r \textrm{d}r \textrm{d}z \right. \\ & \quad \Bigg / \left. \int _{\Omega ^m} \left( | u^{exact}|^2 + | {{\,\textrm{grad}\,}}u^{exact}|^2\right) r \textrm{d}r \textrm{d}z \right) ^{1/2}, \end{aligned}$$

where *u* is a place holder to denote an appropriate scalar field and $e(u) = u^{{exact}}- u^{hp}$ with $u^{hp}$ being the approximate solution for a mesh of size *h* and elements of order *p*.

#### Thermally conducting sphere in a uniform temperature gradient

This problem consists of a sphere of radius $a=1$ m placed in a temperature gradient that is uniform far from the object and at this location is of the form $-G_0{\varvec{e}}_z$ with $G_0=1$. The sphere is thermally conducting and has a different relative thermal conductivity $\kappa _r=100$ compared to the unit background. For this static decoupled problem, the temperature transmission problem (21j-l) reduces to a Poisson equation with associated boundary and transmission conditions and, by analogy to a dielectric sphere in a uniform static electric field, this problem has an analytical solution [, p.g 110–116] in spherical coordinates. This can be expressed in cylindrical coordinates as^13^

35 $$\begin{aligned} T^{{exact}} = {\left\{ \begin{array}{ll} -\left( \frac{3}{\kappa _r + 2}\right) G_0 z & \text { for } \sqrt{r^2 + z^2} < a, \\ -G_0 z + \frac{\kappa _r - 1}{\kappa _r +2}\frac{G_0 a^3 z}{(r^2 + z^2)^{3/2}} & \text { for } \sqrt{r^2 + z^2} \ge a. \end{array}\right. } \end{aligned}$$

Due to the rotationally symmetric nature of this geometry and solution, it can be modelled as an axisymmetric problem. We consider a truncated domain $\Omega ^m = \{ (r,z): \left( 0 \le r \le 2, -2 \le z \le 2\right) \}\text { m}^2$ where the boundary condition $T = T^{{exact}}$ is applied only on three of the boundaries in this axisymmetric setting and the boundary condition on the radial axis is zero Neumann.

**Fig. 4:**
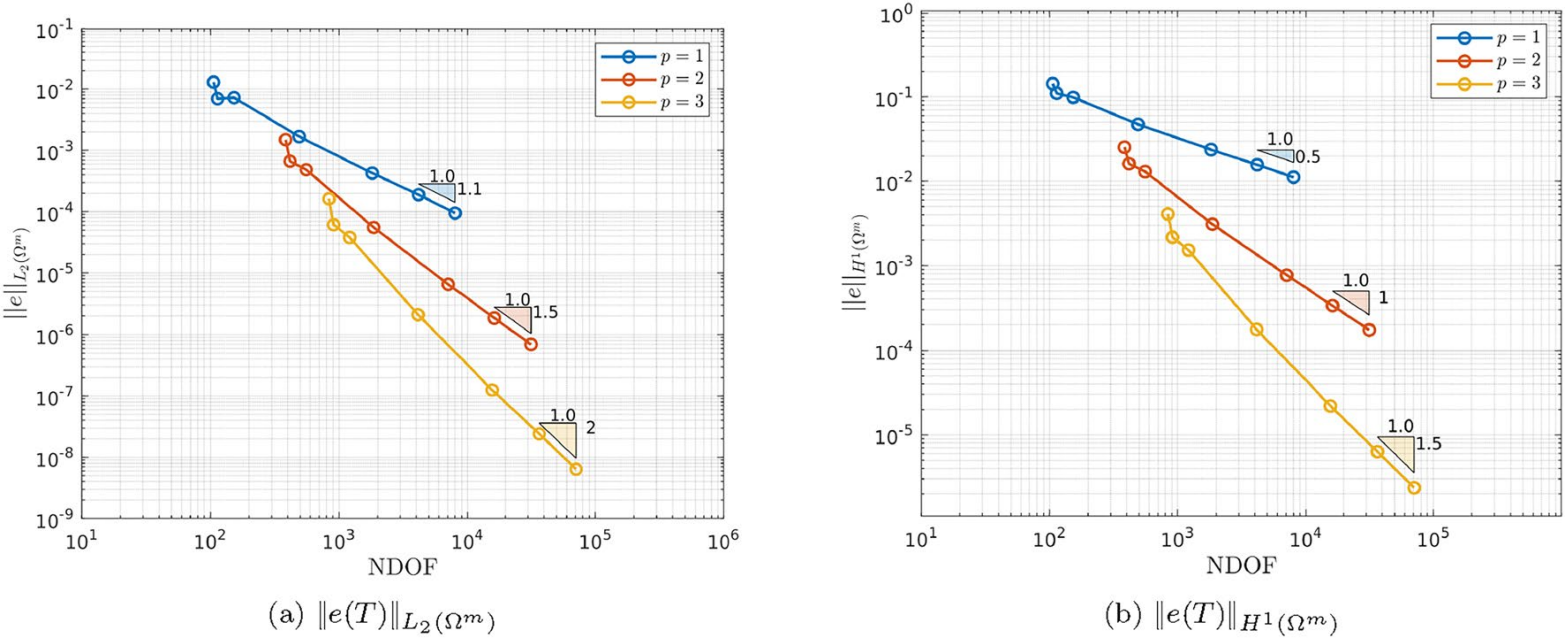
Thermally conducting sphere in a uniform temperature gradient: Convergence of **a** $\Vert e(T)\Vert _{L_2(\Omega ^m)}$ and **b** $\Vert e(T)\Vert _{H^1(\Omega ^m)}$ with respect to *h*-refinement at $p=1,2,3$

As this problem is smooth, it is expected that $\Vert e(T )\Vert _{L_2(\Omega ^m)}$ and $\Vert e(T)\Vert _{H^1(\Omega ^m)}$ will achieve convergence rates of $(p+1)/2$ and *p*/2 with respect to the number of degrees of freedom (NDOF) under uniform *h*-refinement for fixed *p* if shown on a log-log plot [28]. Plots of example meshes for this example can be found in [17], which use curved elements to represent the curved sphere-background interface. Fig. 4 shows that, after a short pre-asymptotic region, the convergence behaviour is algebraic and the slope triangles indicate that the correct rates of convergence are obtained.

**Fig. 5:**
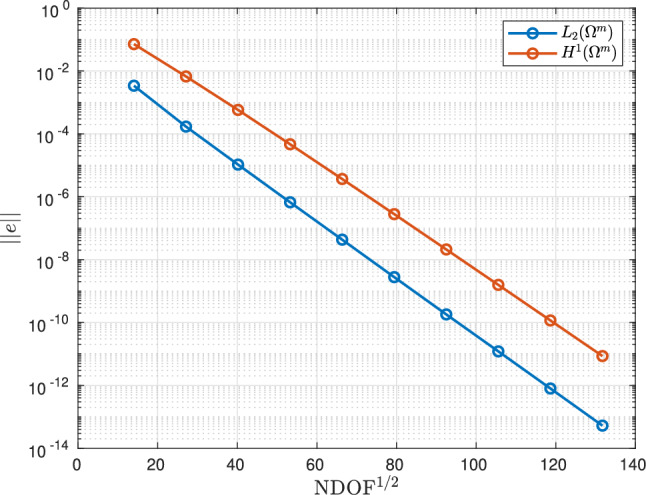
Thermally conducting sphere in a uniform temperature gradient: Convergence of $\Vert e(T)\Vert _{L_2(\Omega ^m)}$ and $\Vert e(T)\Vert _{H^1(\Omega ^m)}$ with respect to *p*-refinement in the static thermal sphere

Next, applying *p*-refinement with $p = 1,2,3,4,5,6,7,8,9,10$ and $p = 4,5,6,7,8,9,10$, in turn, to a mesh with quasi-uniform spacing $h=0.25$ m, the resulting convergence of $\Vert e(T )\Vert _{L_2(\Omega ^m)}$ and $\Vert e(T)\Vert _{H^1(\Omega ^m)}$ with respect to $\hbox {NDOF}^{1/2}$ on a semi-log plot is shown in Fig. 5. The resulting straight lines on this plot indicate that the convergence is exponential with respect to $\text {NDOF}^{1/2}$ and agrees with the a priori convergence rates [28].

**Fig. 6:**
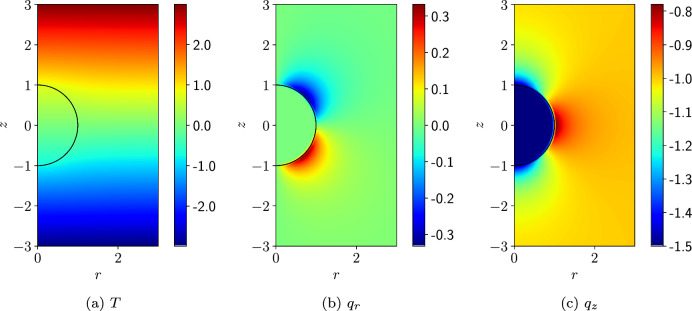
Thermally conducting sphere in a uniform temperature gradient: Contour plots of the **a** *T* field **b** *r*-component of $\varvec{q}$ and **c** the *z*-component of $\varvec{q}$

An illustration of the converged solution is shown in Fig. 6, which shows the solution for *T* as well as the components of the heat flux $\varvec{q}= -\kappa {{\,\textrm{grad}\,}}T$ obtained using $h=0.05$ m and $p=4$ elements. This figure illustrates the smooth solution for *T* and that ${\varvec{q}}$ is discontinuous at the sphere-background interface with ${\varvec{q}}$ being uniform inside the sphere, as expected.

#### Time harmonic conducting sphere in uniform amplitude magnetic field

This problem consists of a conducting magnetic sphere $\Omega _c$ of radius $a = 1$ m with conductivity $\gamma _c = 1 \times 10^7 \text{ S/m}$ and permeability $\mu _c=\mu_0$ placed in a vacuum with a uniform amplitude time varying magnetic field $\varvec{H}_0$ far from the object. Assuming that the magnetic field is sinusoidal with angular frequency $\omega$, then the magnetic field and, hence the vector potential, can be expressed in terms of complex amplitudes that are spatially varying. This means that $\varvec{A}(\varvec{x}, t) = \Re \left( {\pmb {\mathcal {A}}}e^{\mathrm{{i}} \omega t}\right)$ where ${\pmb {\mathcal {A}}}$ is the complex amplitude of $\varvec{A}(\varvec{x},t)$, $\text {i}:= \sqrt{-1}$, and we set ${\pmb {\mathcal {H}}}_0 = |\varvec{B}_0|\mu _0^{-1} \varvec{e}_z$ Wb. In this case, $\varvec{A}(\varvec{x}, t)$ satisfies a simplified version of (21a–21d) with no thermal or circuit coupling. An analytical solution in spherical coordinates is given by [, pages 396–399], however, we can express it in cylindrical coordinates 26$(r,\phi ,z)$ as

36 $$\begin{aligned} {\pmb {\mathcal {A}}}^{{exact}} = {\left\{ \begin{array}{ll} \frac{1}{2}\mu _0^{-1} C |\varvec{B}_0|\left( r^2+z^2\right) ^{-3/4}r I_3 \varvec{e}_\phi & \text { for } \sqrt{r^2 + z^2} < a \\ \frac{1}{2}\mu _0^{-1} |\varvec{B}_0|\left( r + \frac{D r}{(r^2+z^2)^{3/2}}\right) \varvec{e}_\phi & \text { for } \sqrt{r^2 + z^2} \ge a, \end{array}\right. } \end{aligned}$$

where *C* and *D* are expressed as

37a $$\begin{aligned} C&= \frac{3 \mu _c v a^{3/2}}{(\mu _c - \mu _0)vI_1 + \left( \mu _0(1+v^2) - \mu _c\right) I_2}, \end{aligned}$$

37b $$\begin{aligned} D&= \frac{\left( (2\mu _c + \mu _0)v I_1 - \left( \mu _0(1+v^2)+2\mu _c \right) I_2\right) a^3}{(\mu _c - \mu _0)vI_1 + \left( \mu _0(1+v^2) - \mu _c \right) I_2}, \end{aligned}$$

with $I_1 := \sqrt{2\pi /v}\sinh (v)$, $I_2 := \sqrt{2\pi /v}\cosh (v)$, $I_3 := \sqrt{2\pi /v_1}(\cosh (v_1)-1)\sinh (v_1)/v_1$, $v:=\sqrt{\text {i}\gamma _c \omega \mu _c}a$ and $v_1:=\sqrt{\text {i}\gamma _c \omega \mu _c}\sqrt{r^2+z^2}$. We set the background field to be such that $|\varvec{B}_0|=1$ T.

To model this problem numerically, the unbounded domain is truncated a finite distance away from $\Omega _c$ to create the finite computational domain $\Omega$. The boundary $\partial \Omega$ is placed a finite distance from $\Omega _c$ and the exact solution is imposed through the boundary condition $\varvec{n} \times {\pmb {\mathcal {A}}}= \varvec{n} \times {\pmb {\mathcal {A}}}^{{exact}}$. The problem is rotationally symmetric and will be modelled by considering it as an axisymmetric problem on a meridian plane. The resulting truncated domain $\Omega ^m$ is defined as $\Omega ^m = \{ (r,z): \left( 0 \le r \le 4, -4 \le z \le 4\right) \}\text { m}^2$. The exact boundary conditions are applied only on three of the boundaries in this axisymmetric setting and the boundary condition on the radial axis is a zero Neumann condition. A simplified version of the weak form presented (26a) is solved using *hp*-finite elements, where we will solve for ${\hat{{\mathcal {A}}}}_\phi = {\mathcal {A}}_\phi /r$, where ${\mathcal {A}}_\phi$ is the azimuthal component of ${\pmb {\mathcal {A}}}$ in cylindrical coordinates in order to avoid the issues of singularities in 1/*r* as $r \rightarrow 0$.

**Fig. 7:**
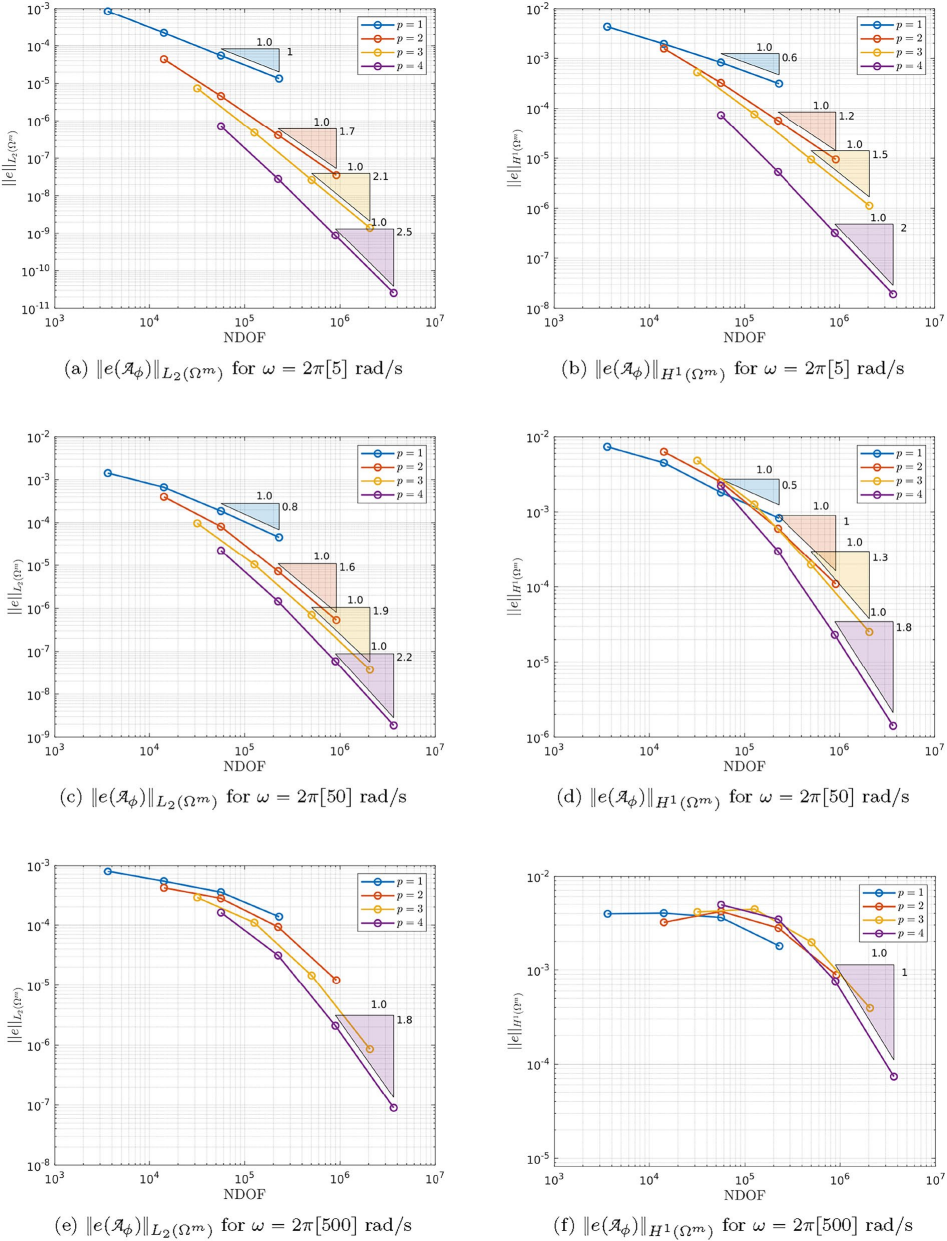
Time harmonic conducting sphere in constant amplitude time varying magnetic field: Convergence of** a**,** c**,** e** $\Vert e({\mathcal {A}}_\phi )\Vert _{L_2(\Omega ^m)}$ and** b**,** d**,** f** $\Vert e({\mathcal {A}}_\phi )\Vert _{H^1(\Omega ^m)}$ with respect to *h*-refinement for $p=1,2,3,4$ at $\omega = 2 \pi [5, 50, 500]$ rad/s

As $\omega$ increases, the skin depth $\delta = \sqrt{2/( \omega \mu _c \gamma _c)}$, which measures the depth to which the Ohmic currents decay to 1/*e* of their surface value, becomes small and the solution to the problem transitions from smooth to having steep gradients just inside the sphere. We illustrate this by plotting $\Vert e({\mathcal {A}}_\phi )\Vert _{L_2(\Omega ^m)}$ and $\Vert e({\mathcal {A}}_\phi )\Vert _{H^1(\Omega ^m)}$ against the NDOF on a log-log plot for *h*-refinement on quasi-uniform triangular meshes and consider different element orders $p=1,2,3,4$ and the frequencies $\omega = 2 \pi [5,50,500]$ rad/s in turn. The results are shown in Fig. 7 where the convergence rates are seen to reduce from the expected rates of $(p+1)/2$ and *p*/2, respectively, for a smooth problem [28], and tend to a limit independent of *p* as $\omega$ increases.

**Fig. 8:**
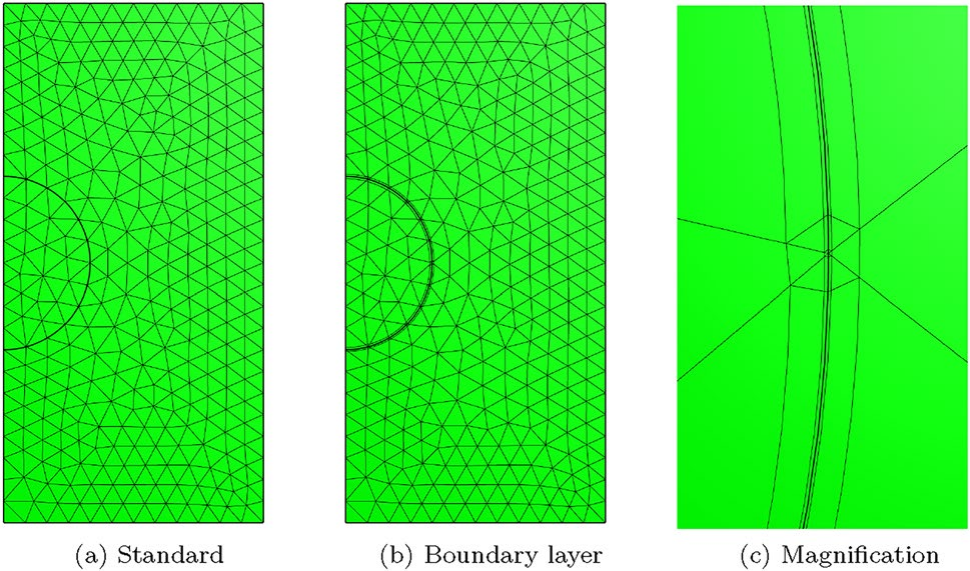
Time harmonic conducting sphere in constant amplitude time varying magnetic field: Illustration of** a** quasi-uniform mesh with average spacing $h=0.25$ m,** b** the same mesh with the addition of two boundary layers and** c** magnification into the boundary layers

**Fig. 9:**
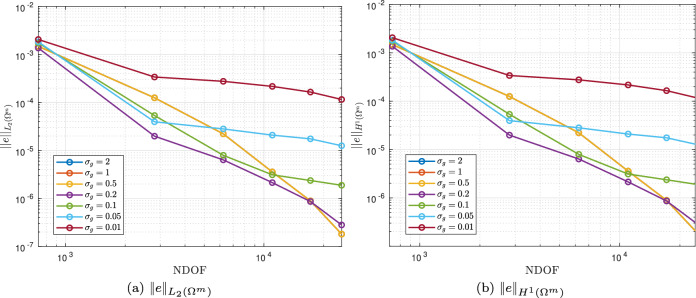
Time harmonic conducting sphere in constant amplitude time varying magnetic field: Convergence of **a** $\Vert e({\mathcal {A}}_\phi )\Vert _{L_2(\Omega ^m)}$ and** b** $\Vert e({\mathcal {A}}_\phi )\Vert _{H^1(\Omega ^m)}$ under *p*-refinement for different $\sigma _g$ for $\omega = 2 \pi [500]$ rad/s and $h=0.25$ m

**Fig. 10:**
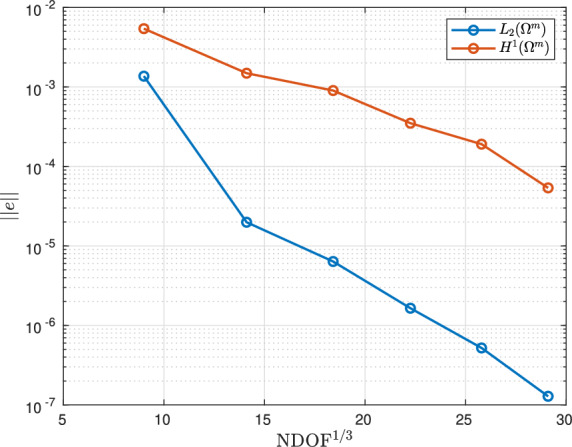
Time harmonic conducting sphere in constant amplitude time varying magnetic field: Convergence of error envelopes of $\Vert e({\mathcal {A}}_\phi )\Vert _{L_2(\Omega ^m)}$ and $\Vert e({\mathcal {A}}_\phi )\Vert _{H^1(\Omega ^m)}$ under *hp*-refinement against $\text {NDOF}^{1/3}$ for $\omega = 2 \pi [500]$ rad/s and $h=0.25$ m choosing the best performing $\sigma _g$ and *hp* layers

**Fig. 11:**
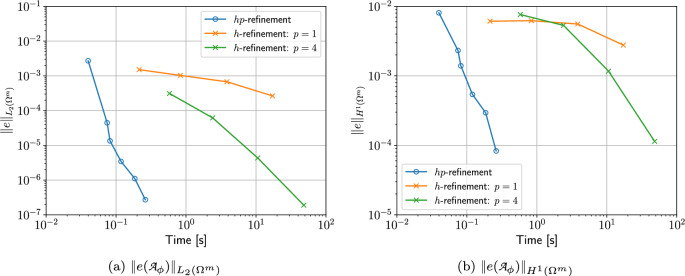
Time harmonic conducting sphere in constant amplitude time varying magnetic field: Convergence of** a** $\Vert e({\mathcal {A}}_\phi )\Vert _{L_2(\Omega ^m)}$ and** b** $\Vert e({\mathcal {A}}_\phi )\Vert _{H^1(\Omega ^m)}$ for $\omega = 2 \pi [500]$ rad/s comparing *hp*-refinement with $h=0.25$ m and the best performing $\sigma _g$ for a mesh with boundary layer, *h*-refinement with uniform $p=1$ elements on a mesh without boundary layers and the same *h*-refinement, but with uniform $p=,4$ elements

To overcome the above issues, we insert quadrilateral elements as boundary layer elements around the surface of the sphere, which, when combined with *p*-refinement, allows us increase the convergence rate and capture the smaller skin depths $\delta$ associated with higher frequencies with less computational effort. In Fig. 8 we show a typical quasi-uniform mesh, a mesh with the addition of two quadrilateral boundary layers and an illustration showing the magnification of the region with boundary layers. By choosing the number of layers and grading factors carefully, the rates of convergence can be further improved. To illustrate this, we consider two layers and consider meshes with a fixed number of 15, 984 triangles with the quadrilateral layers defined according to the different grading factors $\sigma _g = 2,1,0.5,0.2,0.1,0.05,0.01$ and then apply *p*-refinement on each mesh. The results in Fig. 9 show that the best strategy is to combine together *p*- and *h*-refinements corresponding to different orders on different meshes. Then, by choosing the smallest error for each NDOF, and plotting the error envelope against $\text {NDOF}^{1/3}$, the results shown in Fig. 10 are obtained. After an initial pre-asymptotic region, the straight line behaviour of this plot illustrates that using the correct combination of *h*- and *p*-refinements does produce exponential convergence with respect to $\hbox {NDOF}^{1/3}$. Additionally, on Fig. 11, we compare the wall clock times for three refinement strategies on a workstation comprised of a 12-core Intel Xeon W-2265 Processor with 128GB (8x16GB) RAM and a NVIDIA Quadro RTX 4000 8GB graphical processing unit, but only using a maximum RAM use of 500MB. The first strategy uses *hp*-refinement on meshes with boundary layer elements, the second employs uniform *h*-refinement on meshes with uniform $p=1$ elements and the third again employs uniform *h*-refinement, but now with $p=4$ elements. In this figure, we observe the significant benefit in reduction in computer time and accuracy obtained by using the *hp*-refinement strategy.

**Fig. 12:**
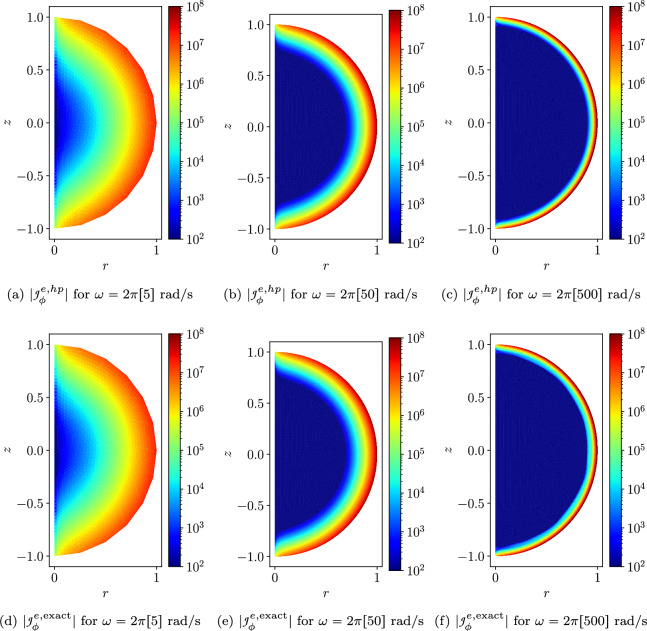
Conducting sphere in a constant amplitude time varying magnetic field: Contour plots with logarithmic scaled color bars of the** a**–**c** $|{\mathcal {J}}^{e,hp}_\phi |$ and** d**–**f** $|{\mathcal {J}}^{e,\text {exact}}_\phi |$ field for $\omega = 2 \pi \left[ 5, 50, 500 \right]$ rad/s with the two layers of quadrilaterals and grading factor $\sigma _g = 0.1$

Figure 12 shows the magnitude of the eddy currents $|{\mathcal {J}}^{e,hp}_\phi |$ for different frequencies obtained for the converged solution compared with the same result for the exact solution. We observe that the thin skin depths are accurately captured and are seen to decrease significantly with increasing frequency.

### Multi-physics quench simulations

The previous single physics examples have motivated the benefits of *p*-refinement over *h*-refinement for a single physics problem with a smooth solution and the benefits of *hp*-refinement with boundary layers for a single physics problem with a steep field gradient due to a material discontinuity. We now consider coupled transient multi-physics quench problems, which are more complicated than the previous single physics benchmarks, but the benefits of *p*-refinement for regions where the solutions are smooth and *hp*-refinement with boundary layers for regions where solutions have steep field gradients immediately carry over to the examples presented below.

#### Single coil

For this benchmark, the coil geometry and proportions of materials follows the example proposed by Wilson [30]. An idealised situation is considered where the coil sits in a large bath of liquid helium, whose extent is truncated far from the coil at a distance of 1 m. The boundary conditions of $T=4.2$ K and ${A}_\phi =0$ are imposed on the truncation boundary. The initial conditions are $I_0=250$ A, $T_0=4.2$ K and $A_\phi (t=0)= r {\hat{A}}_\phi (t=0)$ with ${\hat{A}}_\phi (t=0)$ corresponding to the solution of (25). The quench was assumed to originate from the centre of the coil and start immediately for $t>0$ s, which, instead of prescribing the source $P^{Trigger}$, was achieved by setting a small region of radius 0.0075 m to be conducting. Results are presented for $RRR = 300$ and without the $P^{Hysteresis}$ and $P^{Dyn}$ terms as this is a single coil and cable parameters for these terms are not known.

**Fig. 13:**
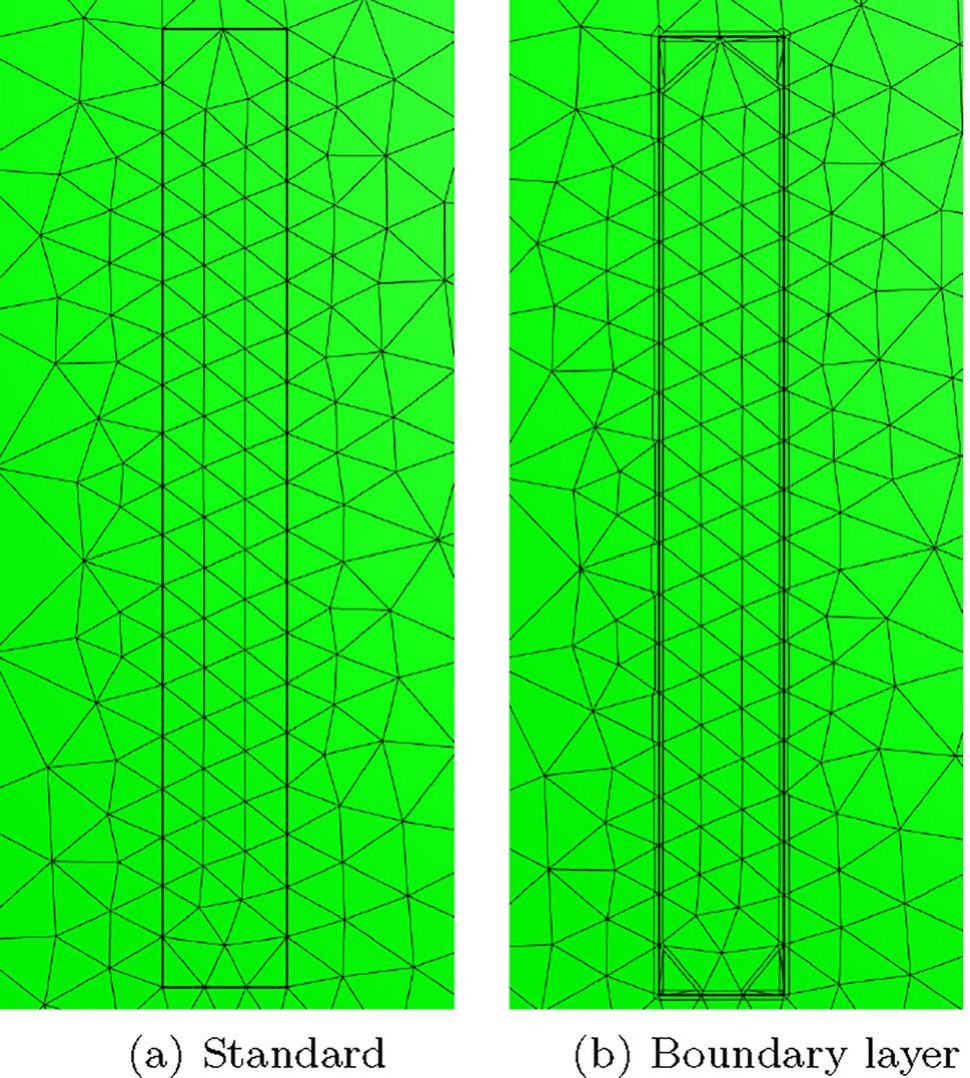
Single coil benchmark problem following Wilson [30]: Showing** a** an unstructured mesh of 684 triangular elements and** b** the same mesh with thin quadrilateral boundary layers included

**Fig. 14:**
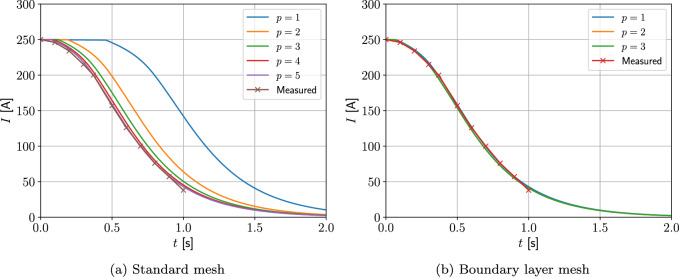
Single coil benchmark problem following Wilson [30]: Showing convergence of *I*(*t*) for** a** *p*-refinement on an unstructured mesh of triangular elements and** b** *p*-refinement on the same mesh with thin quadrilateral boundary layers included

**Fig. 15:**
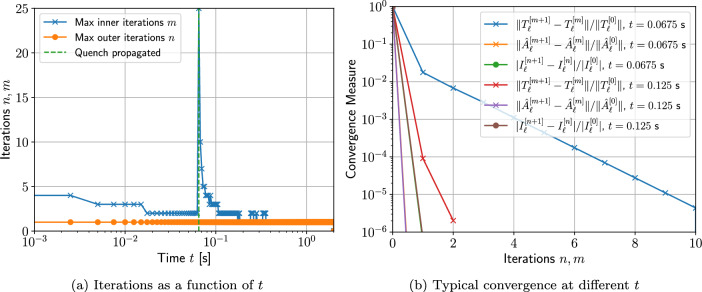
Single coil benchmark problem following Wilson [30]: Showing **a** the number of fixed point iterations required for convergence from Algorithm 1 as a function of *t* and **b** the typical convergence behaviour as a function of number of iterations for several *t* with $TOL = 10^{-4}$

Two different meshes are considered: The first is a coarse mesh of 684 unstructured triangular elements, refined towards the coil, and the second, motivated by the results in Sect. 6.1.2, additionally has a thin layer of quadrilateral elements just inside the coil to model the thin skin-depth effects, resulting in a hybrid mesh with 729 elements in total. The construction of these meshes is shown on Fig. 13. On each, Algorithm 1 is applied using a prescribed time step of $\Delta t = 2.5\times 10^{-3}$ s, which has been checked to be sufficiently small to ensure temporal convergence. On the first mesh, *p*-refinement using order $p=1,2,3,4,5$ elements, in turn, is applied and the resulting behaviour of *I*(*t*) is shown in Fig. 14a. Then, on the second mesh we need to only consider order $p=1,2,3$ elements as this is already sufficient to obtain mesh convergence. The resulting behaviour of *I*(*t*) is shown in Fig. 14b. Also included in Fig. 14 is the measurement data provided by [30], which agrees well with our results. The typical maximum number of iterations as a function of *t* for the inner and outer loops of Algorithm 1 is shown in Fig. 15a, where, other than the time corresponding to when the quench becomes fully propagated over the coil, the number of iterations is small. We remark that this is peak in the number of inner iterations can be reduced by using a small $\Delta t$. In Fig. 15b we highlight the typical convergence behaviour of where we highlight that only a couple of iterations are required to achieve convergence with a smaller number of inner iterations required once the quench has fully propagated over the coil.

**Fig. 16:**
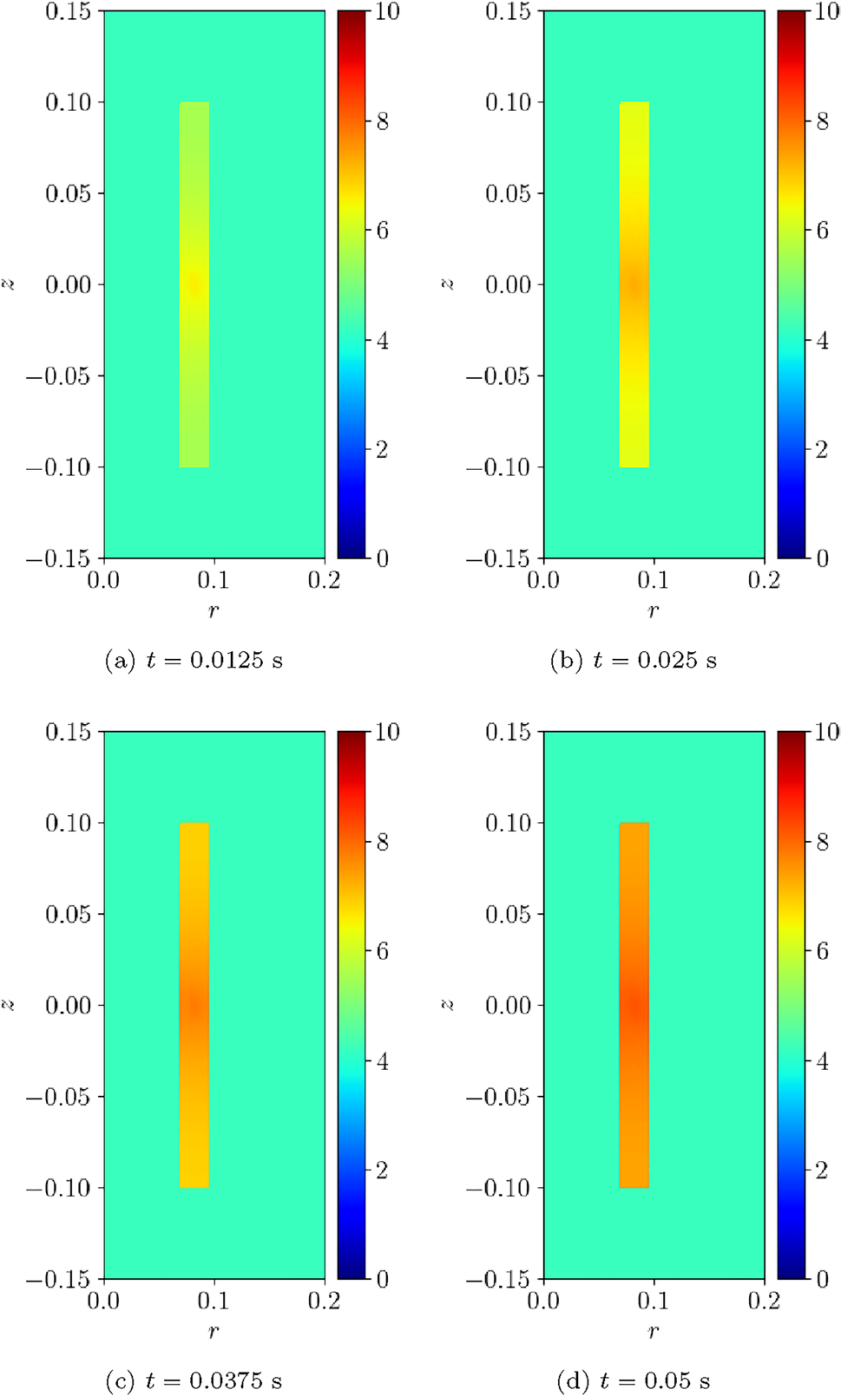
Single coil benchmark problem following Wilson [30]: Illustration of *T*(*t*) obtained at** a** $t=0.0125$ s, **b** $t=0.025$ s,** c** $t=0.0375$ s and** d** $t=0.05$ s

**Fig. 17:**
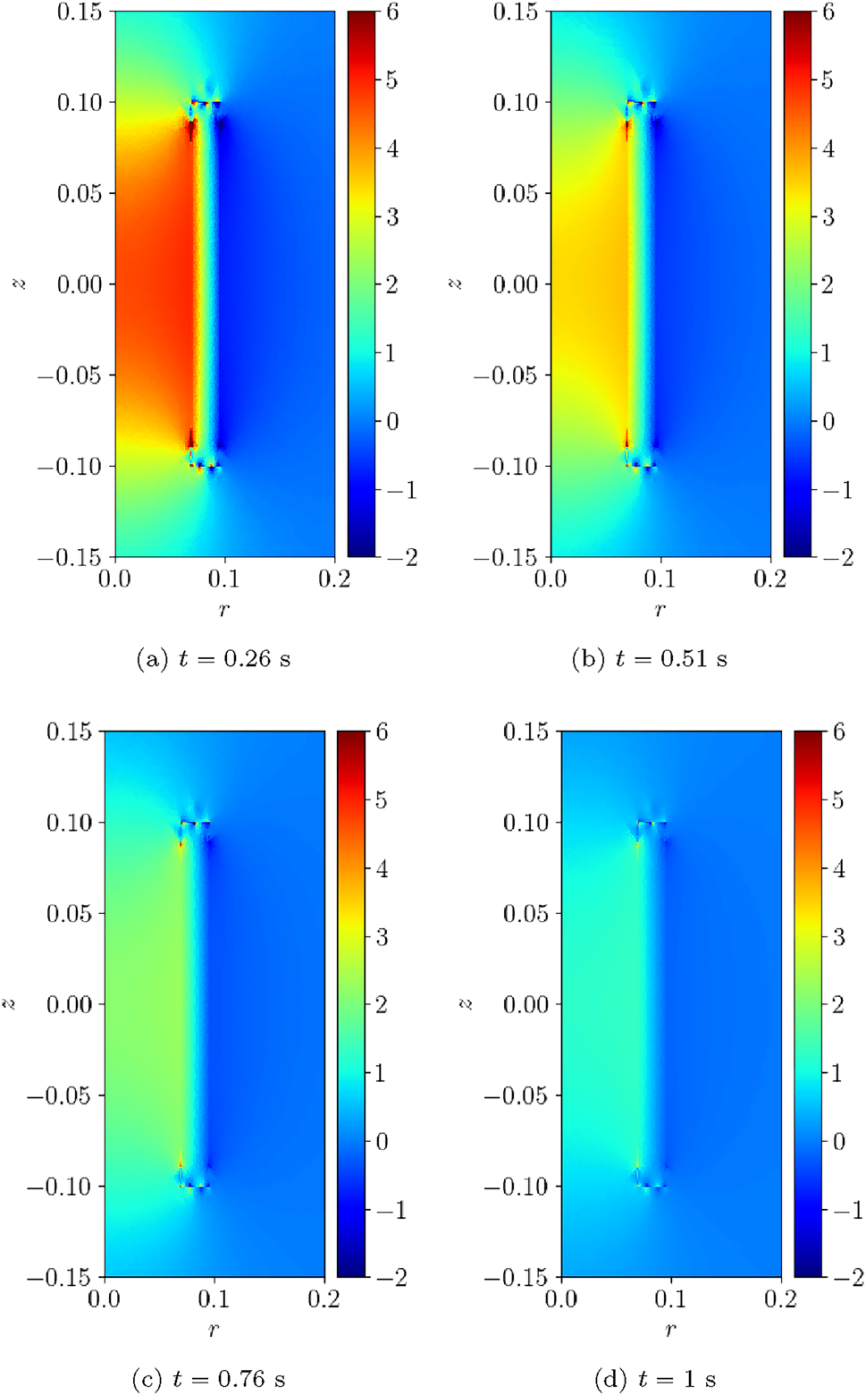
Single coil benchmark problem following Wilson [30]: Illustration of $B_z(t)$ obtained at** a** $t=0.26$ s,** b** $t=0.51$ s,** c** $t=0.76$ s and** d** $t=1$ s

For the converged solution, contours of *T*(*t*) at times $t=0.0125, \, 0.025, \, 0.0375, \, 0.05$ s are shown in Fig. 16a–d, respectively. This figure illustrates that, following initiation of the quench at the centre of the coil, the temperature quickly rises in the form of a shock that propagates outwards from the centre. The solution for $B_z(t)$ at times $t=0.26$, 0.51, 0.76 and 1 s are shown in Fig. 17a–d, respectively. This figure illustrates how the strength of the magnetic flux density decays in line with the current, once the quench has propagated over the coil, and it has become normal conducting.

#### Two coil problem with wire type 1

Next, we consider a two-coil problem. The coils 1 and 2 occupy the regions $\Omega _{s,1}^m=\{(r,z):0.25 \le r \le 0.2884, 0.135\le z \le 0.19473 \}$ $\hbox {m}^2$ and $\Omega _{s,2}^m=\{(r,z): 0.25 \le r \le 0.2884, -0.19473 \le z \le -0.135 \}$ $\hbox {m}^2$, respectively, on the meridian plane. The coils each consist of 986 turns, which gives rise to field a strength of approximately 1.5 T. This can be obtained by re-arranging the Biot-Savart law for an exciting solenoid on axis from [15] or using the ratios of coil cross-section to wire cross-sectional properties. Specifically, wire type 1 is chosen to have the following volume fractions $f_{Cu} =0.6666$, $f_{insul} =0.0437$, $f_{NbTi} = 0.1588$ and $f_{Epoxy} =0.1309$. The self and mutual inductances of each coil are $L=0.8440$ H and $M=0.0873$ H, respectively and are computed from constants of proportionality that arise when relating the total magnetic energy to the current squared, this is explicitly carried out in [17]. The coils are assumed to sit in the idealised situation of a large bath of liquid helium whose extent is truncated far from the coil at a distance of 1 m where the boundary conditions of $T=4.2$ K and $A_\phi =0$ are imposed. Initial conditions are $I_0 = 550$ A, $T_0=4.2$ K and $A_\phi (t=0)= r {\hat{A}}_\phi (t=0)$ with ${\hat{A}}_\phi (t=0)$ corresponding to the solution of (25). The quench was assumed to originate from the location $(r,z)=(0.2501,0.16485)$ m in the upper coil and to start immediately for $t>0$ s, which, rather than prescribing $P^{Trigger}$, was achieved by setting a small region of radius 0.0067 m to be conducting. Note that wire type 1 is additionally chosen to have $RRR=100$, $l_f=0.0375$ m and $a_{sc} = 0.82\times 10^{-3}$ m.

**Fig. 18:**
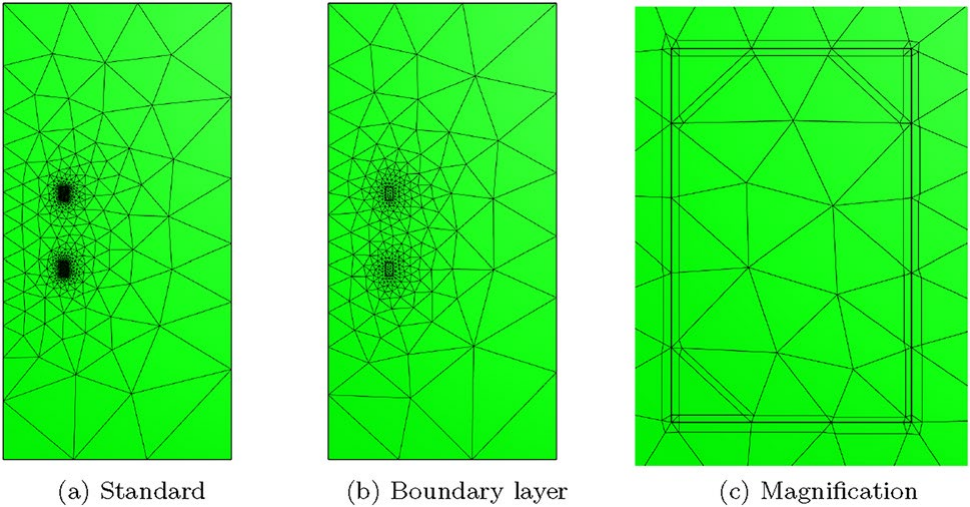
Two coil benchmark problem with wire type 1: Showing** a** an unstructured mesh of 1424 triangular elements refined towards the coil,** b** a coarser mesh with 706 elements, but with two thin quadrilateral boundary layers included and** c** a magnification into the boundary layers

**Fig. 19:**
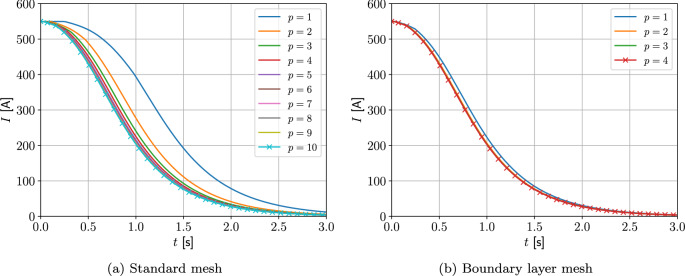
Two coil benchmark problem with wire type 1: Showing convergence of *I*(*t*) for** a** *p*-refinement on an unstructured mesh of triangles** b** *p*-refinement on the same mesh with thin quadrilateral boundary layers included

**Fig. 20:**
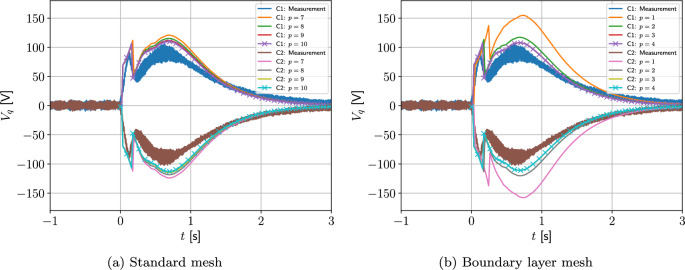
Two coil benchmark problem with wire type 1: Showing convergence of $V_q (t)$ for coils 1 & 2 (C1 & C2)** a** *p*-refinement on an unstructured mesh of triangles** b** *p*-refinement on the same mesh with thin quadrilateral boundary layers included

Two different meshes are considered: The first is a coarse mesh of 1424 triangular elements, refined towards the coils and the second, again motivated by the results in Sect. 6.1.2, uses a thin layer of quadrilateral elements just inside the coil to model the thin skin-depth effects and has 706 elements. The construction of these meshes is shown in Fig. 18. On each, a time step of $\Delta t = 2.5\times 10^{-3}$ s is prescribed, which has been checked to be sufficiently small for temporal convergence. On the first mesh, *p*-refinement is considered with $p=1,2,3,4,5,6,7,8,9,10$, in turn, and while the current *I*(*t*) shown in Fig. 19a rapidly converges, the solution for the quench voltage, which is of the form

$$\begin{aligned} V_{q,n}(t) = I(t)R_n(t) + (L_n+M_n) \frac{\textrm{d}I}{\textrm{d}t}, \end{aligned}$$

shown in Fig. 20a converges much more slowly. On the second mesh, *p*-refinement with $p=1,2,3,4$ is considered and the convergence of the solution for both *I*(*t*) shown in Fig. 19b and the corresponding quench voltage shown in Fig. 20b is rapid. In particular, while the solutions with $p=1$ are inaccurate, the solutions for $p\ge 2$ are seen to converge rapidly and are converged for $p=3$.

**Fig. 21:**
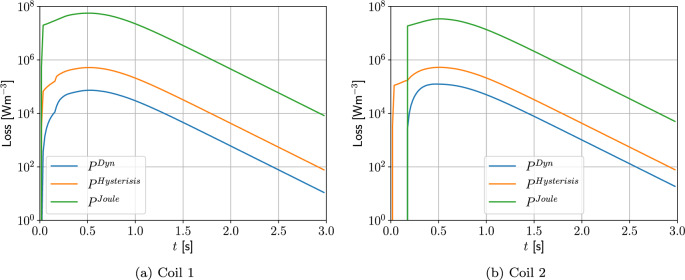
Two coil benchmark problem with wire type 1: Showing the time evolution of the volume averaged contributions of $P^{Dyn}$, $P^{Joule}$ and $P^{Hysteresis}$ when integrated over **a** coil 1 and** b** coil 2

The volume averaged loss contributions $P^{Dyn}$, $P^{Joule}$ and $P^{Hysteresis}$ as a function of time for each of the two coils are shown in Fig. 21. This figure illustrates that $P^{Joule}$ is the dominant contribution for coil 1, which immediately begins to quench for $t>0$ s and is fully quenched shortly afterwards. For coil 2, $P^{Hysteresis}$ provides an important contribution to the heating of the coil and causes it to quench. Once it quenches, $P^{Joule}$ becomes the dominant contribution. The contribution of $P^{Dyn}$ is much smaller.

**Fig. 22:**
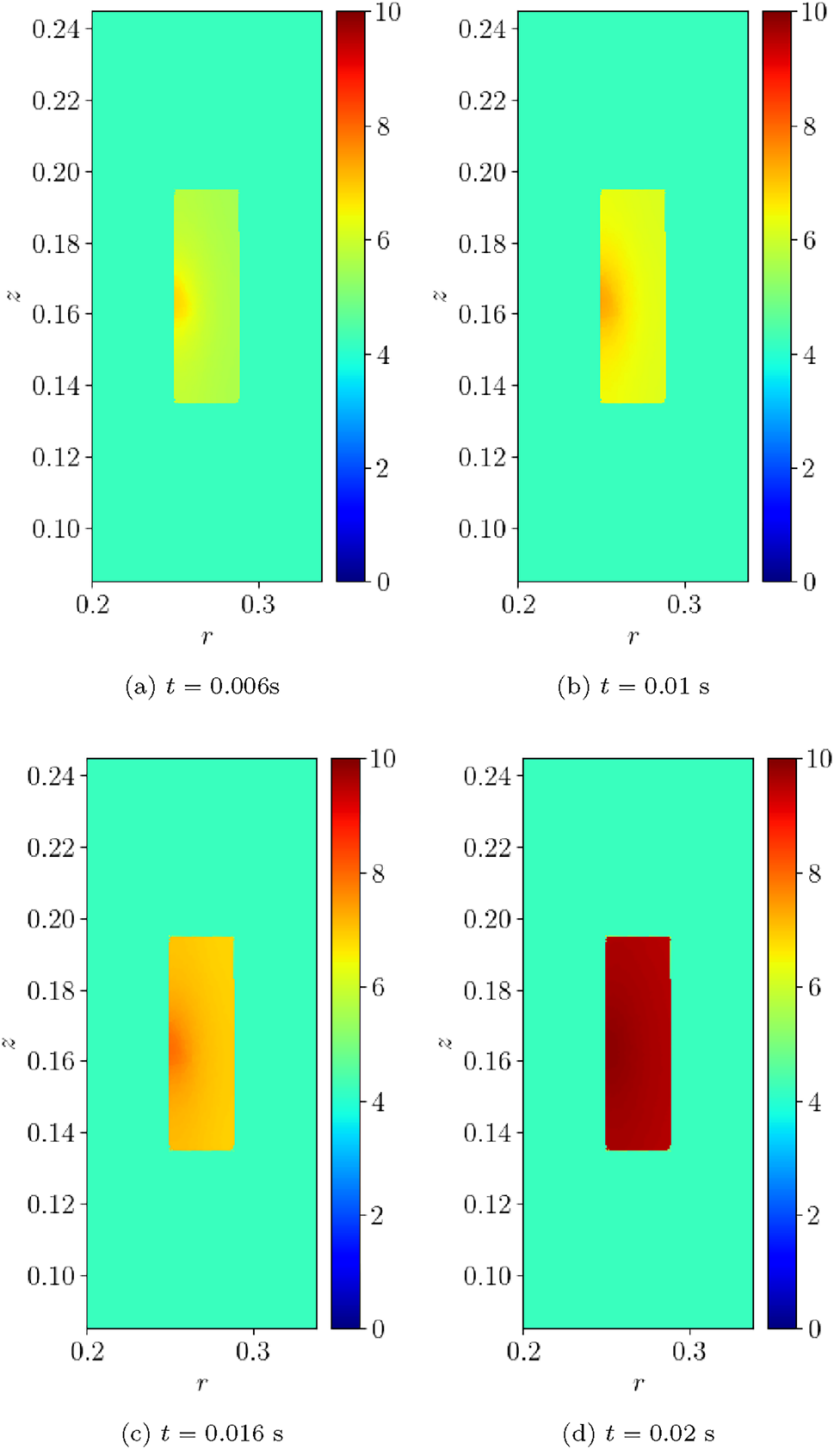
Two coil benchmark problem with wire type 1: Illustration of *T*(*t*) obtained at** a** $t=0.006$ s,** b** $t=0.01$ s,** c** $t=0.016$ s and** d** $t = 0.02$ s in coil 1

**Fig. 23:**
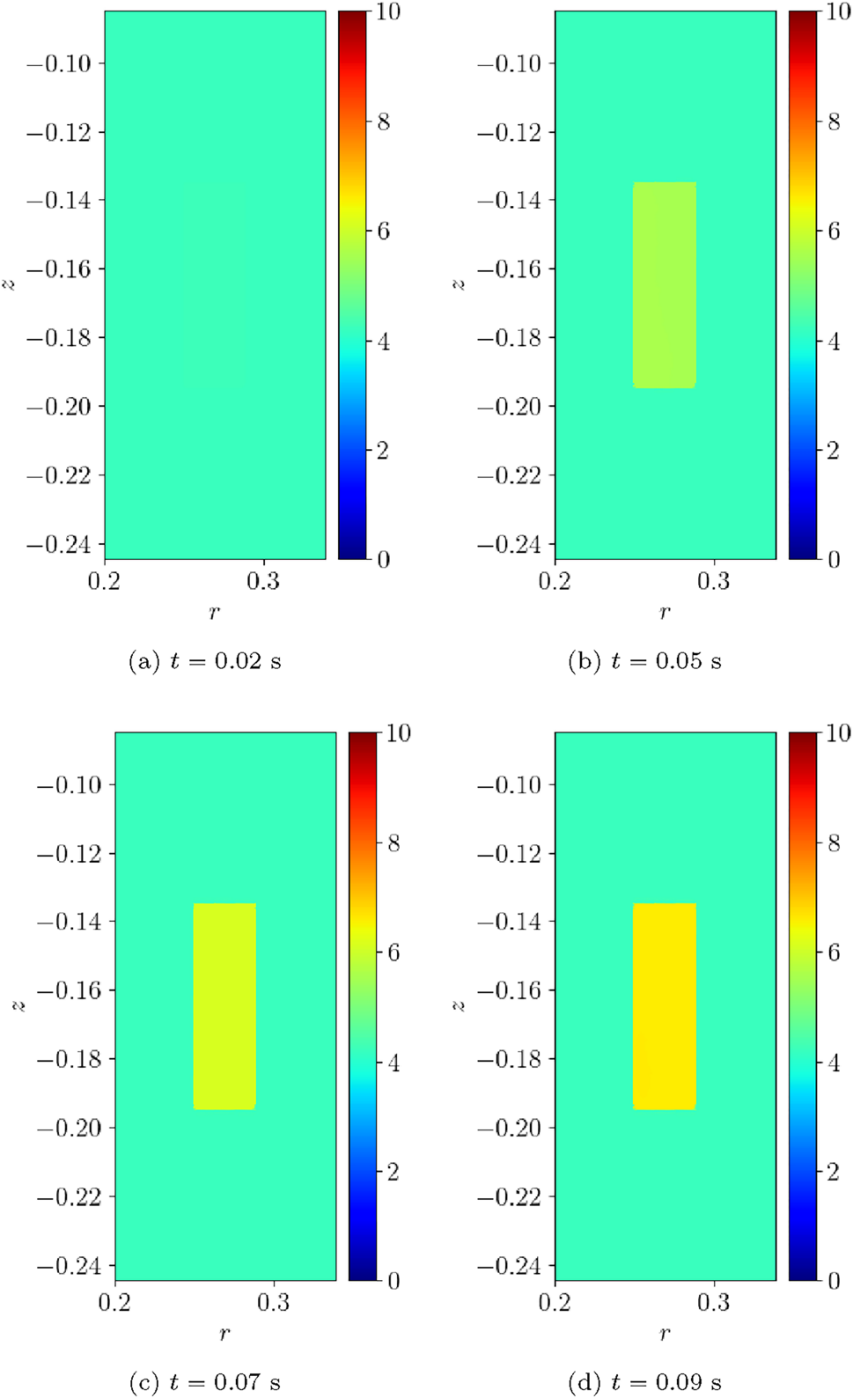
Two coil benchmark problem with wire type 1: Illustration of *T*(*t*) obtained at** a** $t=0.02$ s,** b** $t=0.05$ s,** c** $t=0.07$ s and** d** $t=0.09$ s in coil 2

**Fig. 24:**
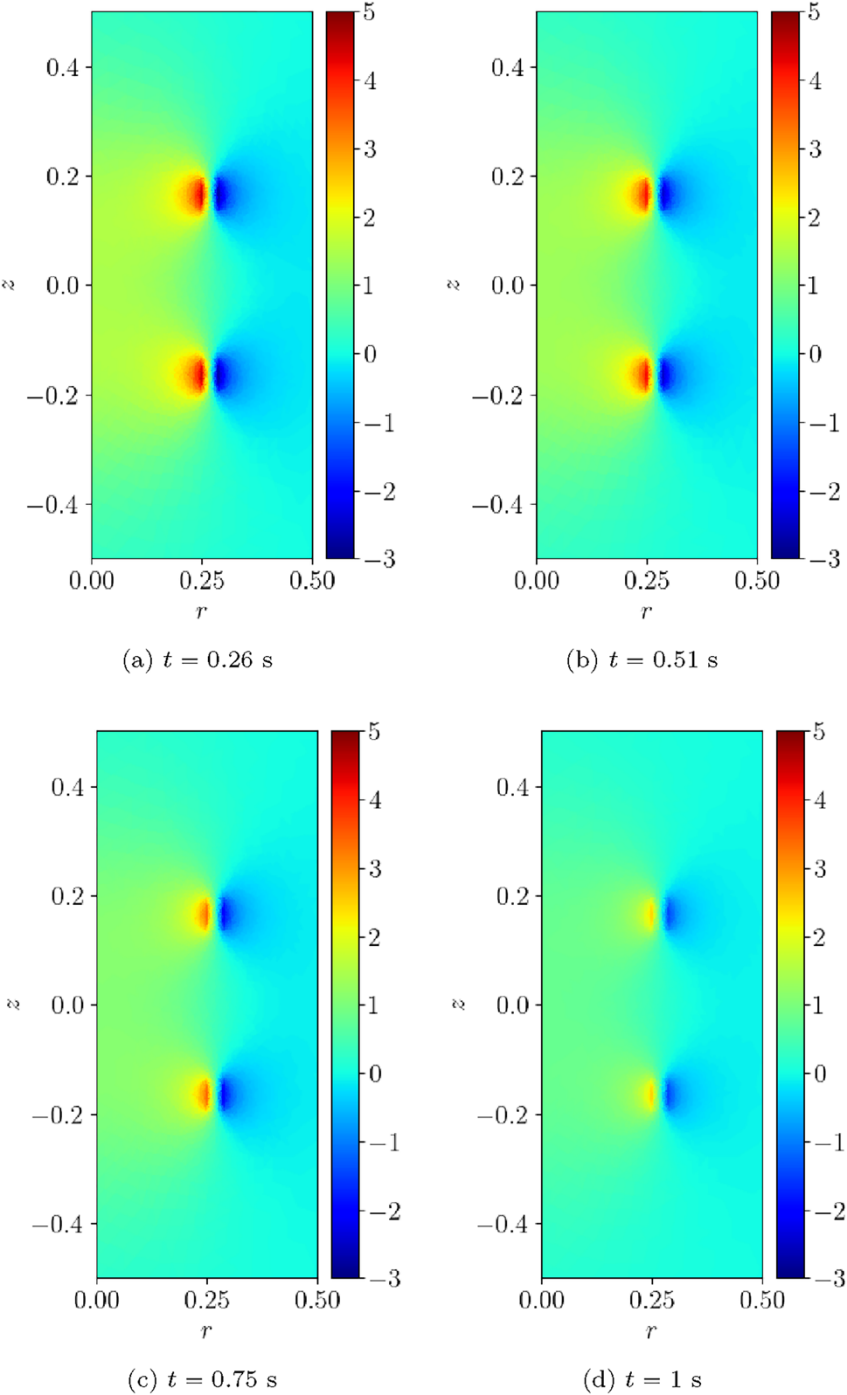
Two coil benchmark problem with wire type 1: Illustration of $B_z(t)$ obtained at** a** $t=0.26$ s,** b** $t=0.51$ s,** c** $t=0.76$ s and** d** $t=1$ s

This is further explained by considering contours of *T*(*t*) in coil 1 at times $t= 0.006, \, 0.01, \, 0.016, \, 0.02$ s shown in Fig. 22a–d, respectively. This shows how the quench quickly propagates in the form of a shock outwards from its initialisation at $(r,z)=(0.2501,0.16485)$ m so that the coil becomes normal conducting. After which, according to Fig. 21, $P^{Joule}$ becomes the dominant heating mechanism. Figure 23a–d shows the much slower heating of the coil 2 at times $t=0.02, \, 0.05, \, 0.07, \, 0.09$ s, respectively, which is also much more homogeneous nature and, according to Fig. 21, occurs due to the $P^{Hysteresis}$ loss before it becomes normal conducting. This coil becomes normal conducting at approximately $t=0.16$ s once the triple $(|{\varvec{B}}|, T, I)$ lies above the critical surface at all points in the coil and then $P^{Joule}$ becomes the dominant heating mechanism for this coil also. Figure  24a–d shows $B_z(t)$ at times $t=0.26$, 0.51, 0.76 and 1 s, respectively, and illustrates the decay of the magnetic flux density once both coils have quenched.

Next, using the aforementioned computer workstation, we compare the simulation time required to obtain the converged solutions, where the simulation used multithreading to exploit the available cores but only used a maximum RAM use of 500MB. The wall clock time to obtain the converged solution with order $p=3$ elements and on the hybrid mesh including the quadrilateral layers was 10 min and 3 s, whereas, without the addition of the layers and with order $p=10$ elements, the wall clock time was 25 min and 13 s. This shows that including the thin quadrilateral layers gives a significant reduction in computational time required to capture the converged solution for the two coil problem.

#### Two coil problem with different wire types

For the next investigation, we consider the performance of different wire types and present the converged results with the finite element model. With the exception of the wire parameters stated in Table 1, the geometry and other details remain unchanged from those given in Sect. 6.2.2.

**Table 1:** Two coil benchmark problem with wire types 1, 2 & 3: Wire types and their parameters

Parameter	Type 1 (T1)	Type 2 (T2)	Type 3 (T3)
$f_{Cu}$	0.66	0.6111	0.7058
$f_{NbTi}$	0.1588	0.2143	0.1483
$f_{Insul}$	0.0437	0.0437	0.0365
$f_{Epoxy}$	0.1315	0.1309	0.1094
$2 a_{sc}$ [m]	$1.64 \times 10^{-3}$	$1.0 \times 10^{-3}$	$1.0 \times 10^{-3}$
*RRR*	100	100	100

**Fig. 25:**
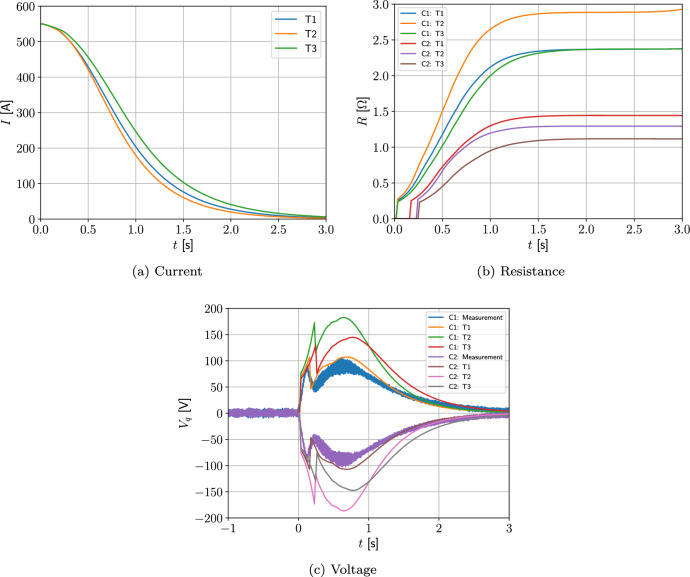
Two coil benchmark problem with wire types 1, 2 & 3 (T1, T2 & T3) and coils 1 & 2 (C1 & C2): Results for** a** *I*(*t*),** b** *R*(*t*) and** c** $V_{q,n}(t)$ obtained with a finite element approach

By again considering the same hybrid mesh of 706 elements, including a quadrilateral layer, and employing *p*-refinement, the converged results for the new wire types were obtained. The results shown in Fig. 25 depict the variation in *I*(*t*), *R*(*t*) and $V_{q,n}(t)$ for the different wire types. Also included is the measurement of $V_{q,n}(t)$ for the two coil problem where the exact wire parameters are not known. It can be observed that the results for type 1 are the closest to the measurements among the the wire types 1, 2 and 3 used.

Note that the wire parameters for wire type 1 and 3 are similar and so the resulting *I*(*t*), *R*(*t*) and $V_{q,n}(t)$ obtained for these wire types are closer to each other compared to the results for wire type 2.

#### Two coil problem with wire type 1 and aluminium formers

**Fig. 26:**
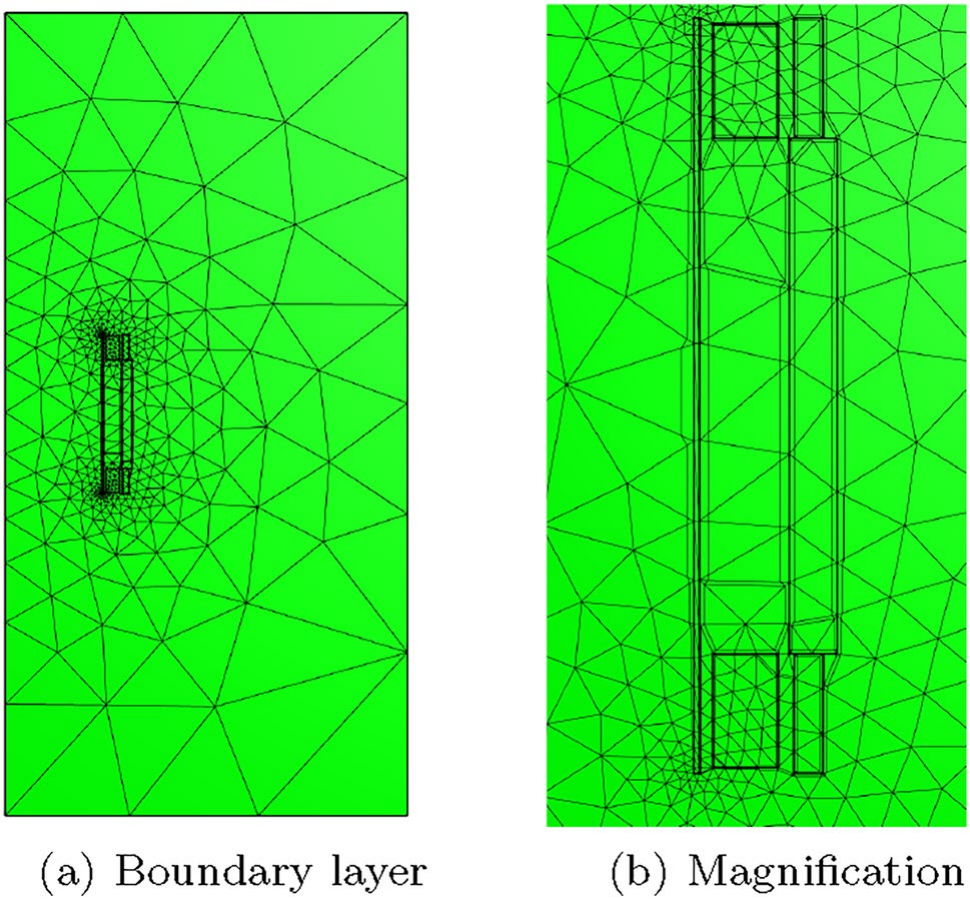
Two coil benchmark problem with formers and wire type 1:** a** The unstructured mesh of triangular elements with quadrilateral layers and** b** magnification of the same mesh

A more realistic model of the two coil problem includes a set of six aluminium formers $\Omega _{c,A_1}^m$, $\Omega _{c,A_2}^m$, $\Omega _{c,B_1}^m$, $\Omega _{c,B_2}^m$, $\Omega _{c,F}^m$ and $\Omega _{c,F_{bore}}^m$ on the meridian plane, these subdomains are all normal conducting and have been chosen to have material properties $\gamma =1 \times 10^7$ S/m and $\mu =\mu _0$. The dimensions of the formers are stated in Table  2. With the exception of the inclusion of the formers, the geometry and other details remain unchanged from those given in Sect. 6.2.2. Again motivated by the results in Sect. 6.1.2, a mesh with 1356 elements, including a layer of quadrilaterals just inside each of the coils and each of the aluminium formers, was generated and is illustrated in Fig. 26.

**Table 2:** Two coil benchmark problem with aluminium formers and wire type 1: Former geometry where each is of the form $\{(r,z):r_1 \le r \le r_2, z_1 \le z \le z_1\}$ $\hbox {m}^2$

$\Omega _c^m$ subdomain	$r_1$ [m]	$r_2$ [m]	$z_1$ [m]	$z_2$ [m]
$\Omega _{c,A_1}^m$	0.2925	0.3075	$-0.198$	$-0.16656$
$\Omega _{c,A_2}^m$	0.2925	0.3075	$-0.16655$	$-0.1351$
$\Omega _{c,B_1}^m$	0.2925	0.3075	0.1351	0.16655
$\Omega _{c,B_2}^m$	0.2925	0.3075	0.16656	0.198
$\Omega _{c,F}^m$	0.29	0.315	$-0.135$	0.135
$\Omega _{c,F_{bore}}^m$	0.24	0.243	$-0.198$	0.198

**Fig. 27:**
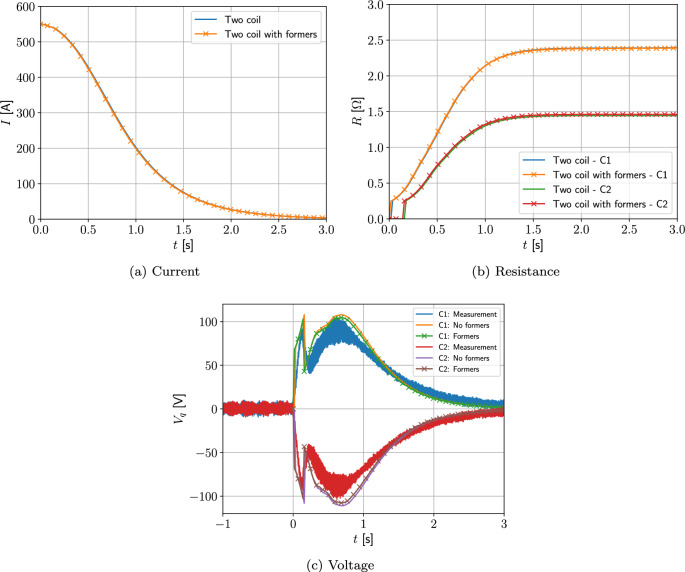
Two coil benchmark problem with formers and wire type 1: Results for coils 1 & 2 (C1 & C2)** a** *I*(*t*),** b** *R*(*t*) and** c** $V_{q,n}(t)$ obtained with a finite element approach for with and without the formers included

While including aluminium formers is expected to modify the ${\varvec{B}}(t)$ field, the changes to the *T*(*t*) field and the circuit quantities *R*(*t*), *I*(*t*) and $V_{q,n}(t)$ are expected to be small as they are driven by the quench effects within the coil. The results provided in Fig. 27 illustrate *R*(*t*), *I*(*t*) and $V_{q,n}(t)$ with and without the formers and it is shown that including the formers results in a small change in $V_{q,n}(t)$, but not any significant differences in the current and resistance.

**Fig. 28:**
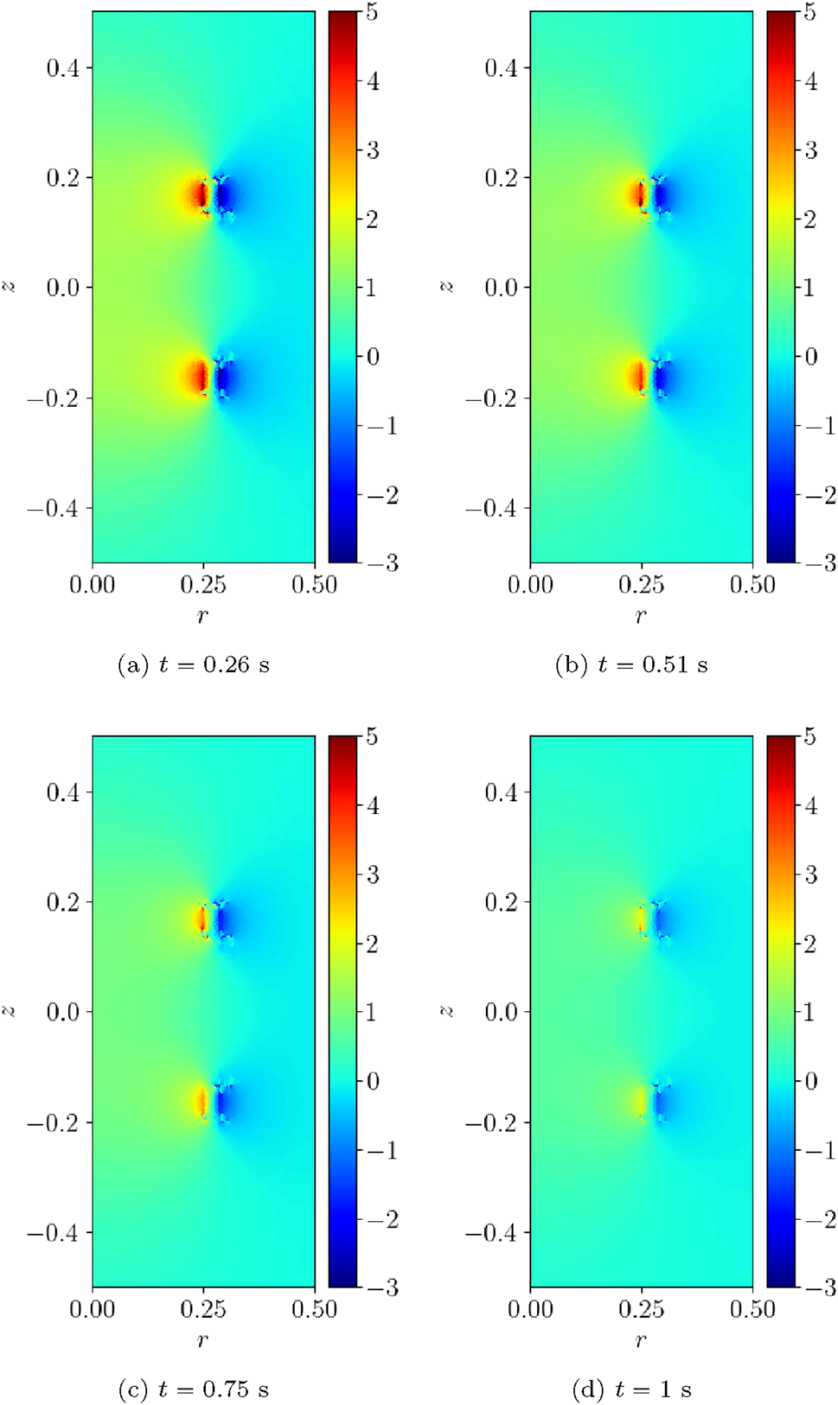
Two coil benchmark problem with formers and wire type 1: Illustration of $B_z(t)$ obtained at** a** $t = 0.25$ s,** b** $t= 0.5$ s,** c** $t= 0.75$ s and** d** $t=1$ s

**Fig. 29:**
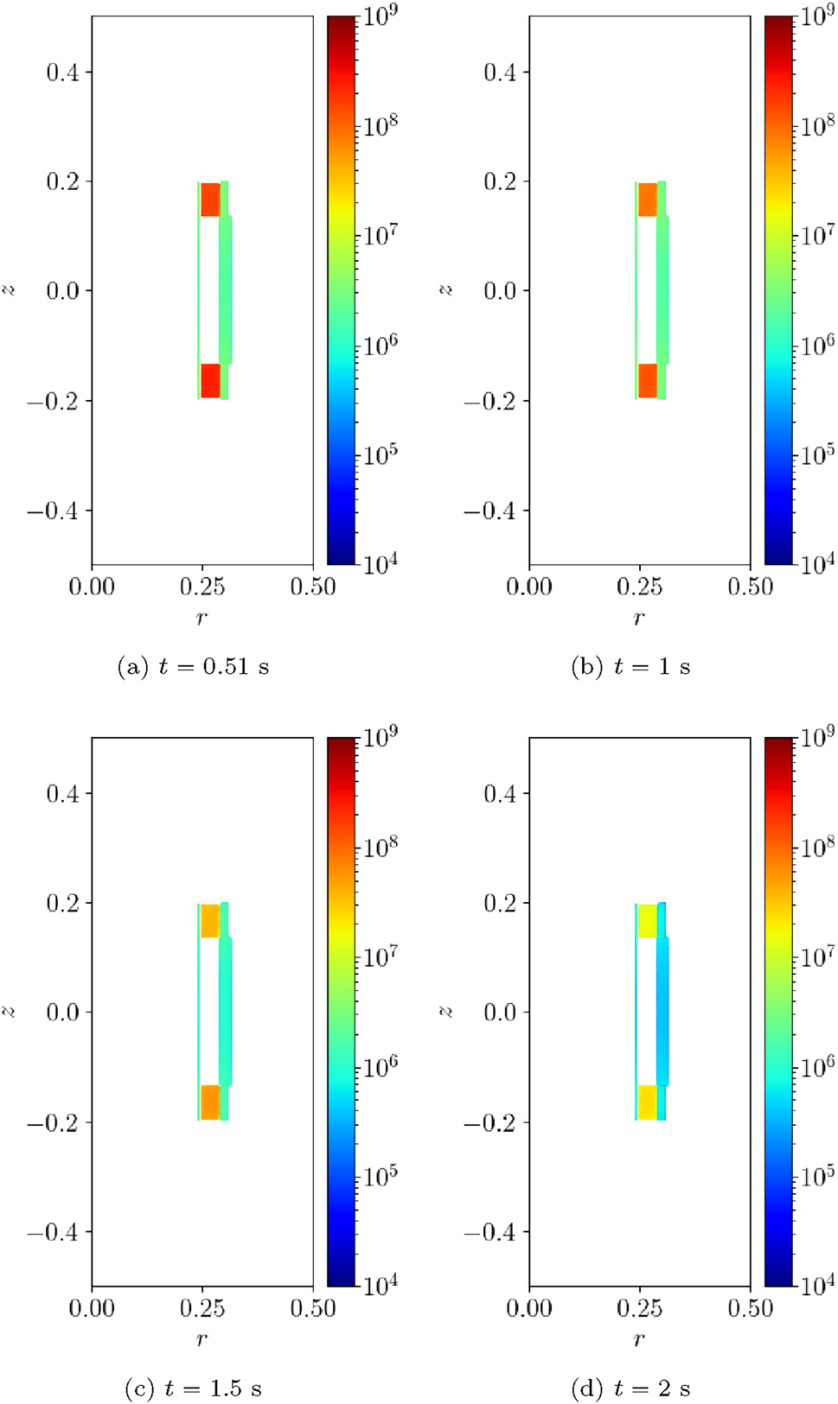
Two coil benchmark problem with formers and wire type 1: Illustration of $|J_\phi ^{e}(t)|$ obtained at** a** $t =0.5$ s,** b** $t= 1$ s,** c** $t= 1.5$ s and** d** $t= 2$ s

The differences in the ${B}_z(t)$ field distribution are illustrated by comparing the results shown in Fig. 28a–d, which include the formers, to those in Figure  24a–d, which do not include the formers. Here the changes in the ${B}_z(t)$ field due to the presence of eddy currents in the formers is clearly observed. It is also of interest to visualise the Ohmic currents that arise in conducting components once the quench begins to propagate, and the electromagnetic fields become time varying. These currents correspond to

38 $$\begin{aligned} \varvec{J}^{e} = \gamma \varvec{E} = J_\phi ^{e} \varvec{e}_\phi = -\gamma \frac{\partial A_\phi }{\partial t} \varvec{e}_\phi , \end{aligned}$$

and are showcased in Fig. 29a–d as a function of *t* for $t= 0.25$, $t= 0.5$, $t= 0.75$ and $t= 1$ s, respectively.

**Fig. 30:**
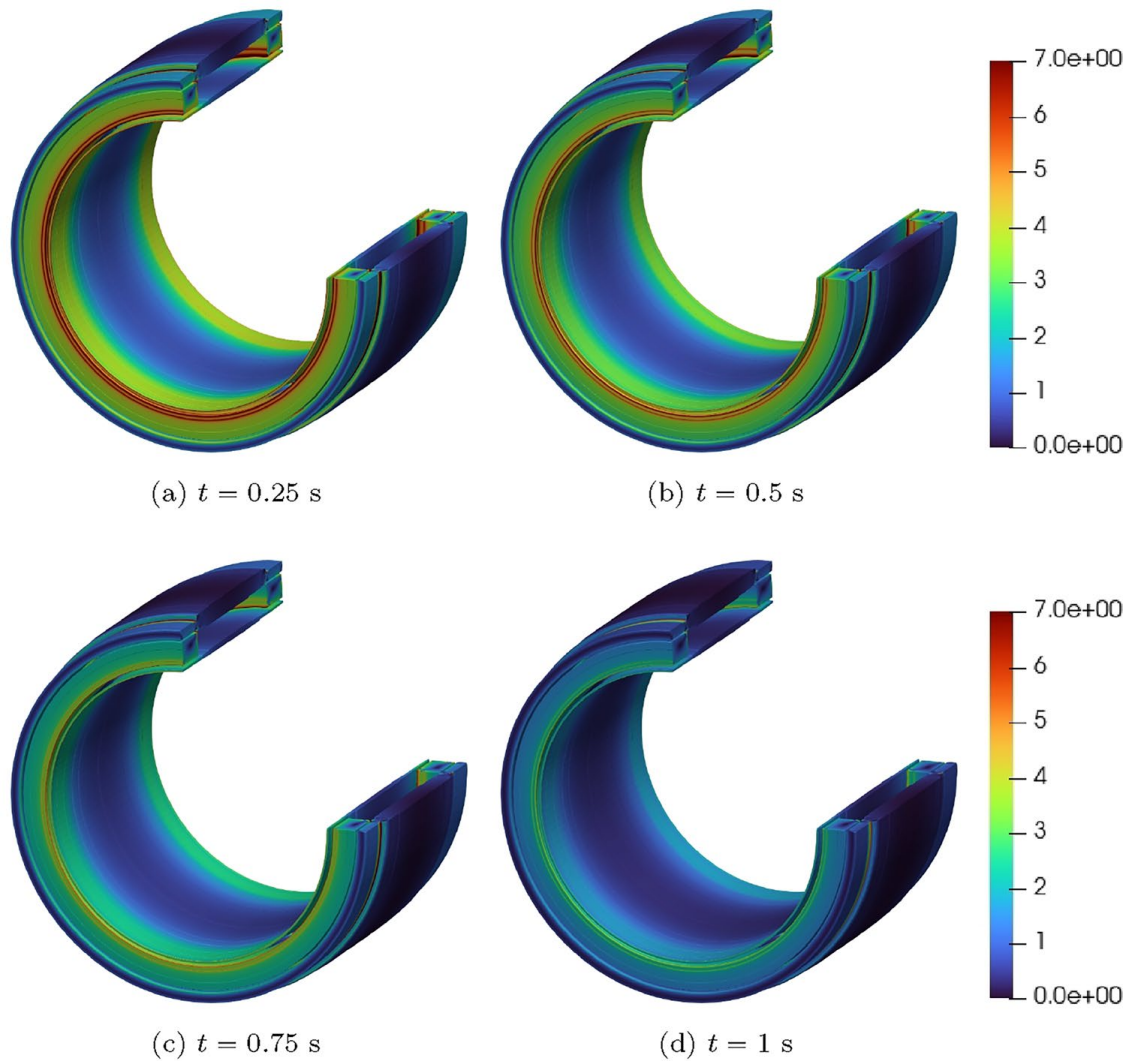
Two coil benchmark problem with formers and wire type 1: 3D Illustration of $|\varvec{B}(t)|$ obtained at (a) $t =0.25$ s, (b) $t= 0.5$ s, (c) $t= 0.75$ s and (d) $t= 1$ s

**Fig. 31:**
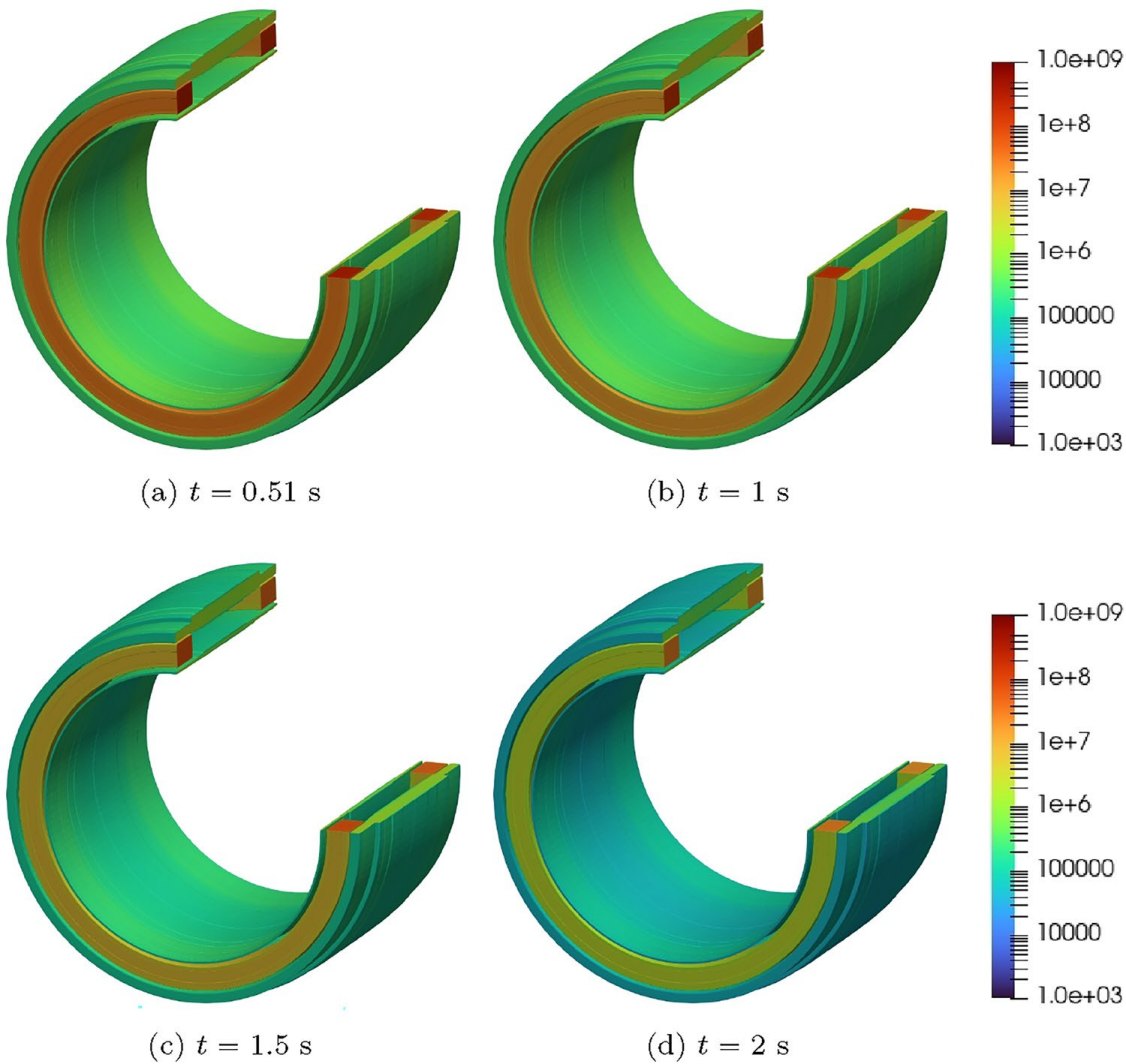
Two coil benchmark problem with formers and wire type 1: 3D Illustration of $|J_\phi ^{e}(t)|$ obtained at** a** $t =0.5$ s,** b** $t= 1$ s,** c** $t= 1.5$ s and** d** $t= 2$ s

Finally, to reiterate the three-dimensional nature of the problem, the solutions for $|\varvec{B}(t)|$ obtained at $t=0.25$, $t=0.5$, $t=0.75$ and $t=1$ s and $|J_\phi ^e(t)|$ obtained at $t=0.51$, $t=1$, $t=1.5$ and $t=2$ s on the meridian plane, have been rotated through $2\pi$ to produce the contour plots shown in Figs. 30a–d and 31a–d, respectively. Our axisymmetric formulation assumes the solution is the same independent of the angular direction $\phi$, which is also emphasised by this plots. However, in reality, the quench would propagate outwards from a point in the angular direction as well as across the (*r*, *z*) plane, which our current model is not able to represent. Nonetheless, the propagation in the angular direction is rapid and our results are in good agreement with the measurements.

#### Two coil with aluminium formers comparison with internal Siemens Healthineers quench modelling software

**Fig. 32:**
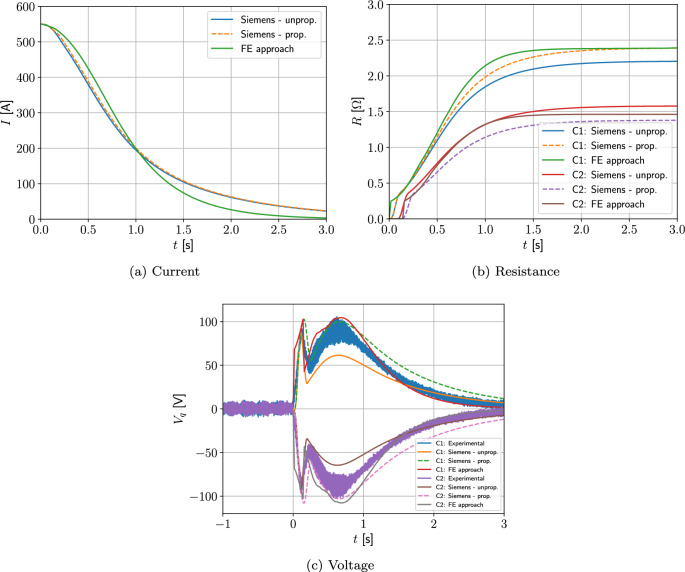
Two coil benchmark problem with formers and wire type 1: Results for coils 1 & 2 (C1 & C2) **a** *I*(*t*),** b** *R*(*t*) and** c** $V_{q,n}(t)$ obtained with a finite element (FE) approach and compared against results obtained with an internal Siemens Healthineers quench modelling software

The solutions for *I*(*t*), *R*(*t*) and $V_{q,n}(t)$ obtained with the finite element model in Sect. 6.2.4 are now compared to an internal Siemens Healthineers quench modelling software for the two coil model with formers. Whilst this is a closed software, it utilises a numerical and analytical combined approach employing a finite difference discretisation. In the Siemens Healthineers software, the quenching of coil 2 through the $P^{Hysteresis}$ is not always guaranteed and so the software additionally activates heating of the second coil using additional $P^{Trigger}$ terms (or identifying regions to be tagged as conducting at certain times during the simulation). The comparison of the converged results obtained by the Siemens Healthineers software for the two coil problem with, and without, these additional terms (propagated and unpropagated) is shown in Fig. 32. We observe that the inclusion of these additional heating terms does not change the current significantly, but improves the agreement of the quench voltage to the measured data for this problem. The resistances generated by the two Siemens Healthineers software models are also slightly different. Comparing the results obtained using the propagated Siemens Healthineers software model and the finite element model, we see the superior agreement of the latter to the experimental data for the quench voltage. This illustrates the importance of accurately capturing the skin depth effects and high field gradients, which is possible using the high order *hp*-version finite element refinements.

## Conclusions

This paper has first presented a review of the key non-linear governing equations and coupling effects involved in MRI magnet quench. The paper has then presented a mathematical model for modelling quench effects using the stranded conductor model and then established a new numerical scheme employing the NGSolve finite element library and a discretisation using high order *hp*-version finite elements including thin quadrilateral elements for resolving boundary layer effects. This scheme has resulted in accurate results for a set of single physics benchmark problems and a set of challenging coupled multi-physics problems involving quench phenomena. In particular, the addition of boundary layer elements and *p*-refinement results in superior convergence properties and accurate resolution of skin depth effects and high temperature gradients and leads to numerical results that are in close agreement with measured data. By including conducting shields, the agreement of the numerical results for a two coil quench problem with the measured data is seen to improve further. The presented computational model has the potential to be applied to more complex quench scenarios and also to include further coupled physics effects, which, together with its extension to three-dimensional problems described in Sect. 5.4, will be the subject of further work. Also, as part of future work, it would be of interest to investigate the application of alternative time integrators once the quench has propagated over the conductor or to use automatic adjustment of the timestep size during the simulations to provide greater computational efficiencies.

## Data Availability

Data sets generated during the current study are available from the corresponding author on reasonable request.
